# Supplementary material for: The sexual experience of Italian adults during the COVID-19 lockdown
Source: PLoS One. 2022 May 5;17(5):e0268079. doi: 10.1371/journal.pone.0268079 (PMC9070892; doi:10.1371/journal.pone.0268079)
Supplement: S1 Table — Frequency information about words extracted from answers to open-ended questions are reported. In the first column. word stems are shown. Frequencies (F(1–5). total frequencies (F(total)). term frequencies (TF(1–5)). inverse document frequencies (IDF) and term-frequency-inverse document frequencies (TF-IDF(1–5)) related to the five context are reported for each term. The last row “Total” shows the measure of the length of each document, where the latter is considered as the total number of stems for each open-ended question multiplied by their frequency (F(1–5)). (DOCX) [file pone.0268079.s001.docx]

**S1 Table. Frequency Information of Total Wordlist extracted from the Answers to Open-Ended Questionnaire**

| ***Stems*** | ***F(1)*** | ***F(2)*** | ***F(3)*** | ***F(4)*** | ***F(5)*** | ***F(total)*** | ***TF1*** | ***TF2*** | ***TF3*** | ***TF4*** | ***TF5*** | ***IDF*** | ***TF-IDF1*** | ***TF-IDF2*** | ***TF-IDF3*** | ***TF-IDF4*** | ***TF-IDF5*** |
| --- | --- | --- | --- | --- | --- | --- | --- | --- | --- | --- | --- | --- | --- | --- | --- | --- | --- |
| abbattuta | 0 | 1 | 0 | 0 | 0 | 1 | 0 | .00071 | 0 | 0 | 0 | 1.60944 | 0 | .00115 | 0 | 0 | 0 |
| abbracci* | 2 | 1 | 0 | 0 | 4 | 7 | .00083 | .00071 | 0 | 0 | .00221 | .51083 | .00042 | .00036 | 0 | 0 | .00113 |
| abbrutimento | 0 | 0 | 0 | 0 | 1 | 1 | 0 | 0 | 0 | 0 | .00055 | 1.60944 | 0 | 0 | 0 | 0 | .00089 |
| accappatoio | 0 | 0 | 0 | 1 | 0 | 1 | 0 | 0 | 0 | .00056 | 0 | 1.60944 | 0 | 0 | 0 | .00090 | 0 |
| accarezz* | 0 | 1 | 0 | 0 | 1 | 2 | 0 | .00071 | 0 | 0 | .00055 | .91629 | 0 | .00065 | 0 | 0 | .00051 |
| accett* | 1 | 4 | 1 | 0 | 7 | 13 | .00041 | .00285 | .00101 | 0 | .00387 | .22314 | .00009 | .00064 | .00022 | 0 | .00086 |
| accolto | 0 | 0 | 0 | 0 | 2 | 2 | 0 | 0 | 0 | 0 | .00111 | 1.60944 | 0 | 0 | 0 | 0 | .00178 |
| accondiscendente | 0 | 1 | 0 | 0 | 0 | 1 | 0 | .00071 | 0 | 0 | 0 | 1.60944 | 0 | .00115 | 0 | 0 | 0 |
| accontento | 0 | 1 | 0 | 0 | 0 | 1 | 0 | .00071 | 0 | 0 | 0 | 1.60944 | 0 | .00115 | 0 | 0 | 0 |
| adoro | 1 | 0 | 0 | 0 | 0 | 1 | .00041 | 0 | 0 | 0 | 0 | 1.60944 | .00066 | 0 | 0 | 0 | 0 |
| affannarmi | 0 | 0 | 0 | 0 | 1 | 1 | 0 | 0 | 0 | 0 | .00055 | 1.60944 | 0 | 0 | 0 | 0 | .00089 |
| affaticamento | 1 | 0 | 0 | 0 | 0 | 1 | .00041 | 0 | 0 | 0 | 0 | 1.60944 | .00066 | 0 | 0 | 0 | 0 |
| affett* | 7 | 1 | 0 | 0 | 8 | 16 | .00289 | .00071 | 0 | 0 | .00443 | .51083 | .00148 | .00036 | 0 | 0 | .00226 |
| affiatamento | 1 | 0 | 0 | 0 | 1 | 2 | .00041 | 0 | 0 | 0 | .00055 | .91629 | .00038 | 0 | 0 | 0 | .00051 |
| afrodisiaca | 0 | 0 | 1 | 0 | 0 | 1 | 0 | 0 | .00101 | 0 | 0 | 1.60944 | 0 | 0 | .00162 | 0 | 0 |
| agedo | 0 | 0 | 0 | 0 | 1 | 1 | 0 | 0 | 0 | 0 | .00055 | 1.60944 | 0 | 0 | 0 | 0 | .00089 |
| aggressiva | 0 | 0 | 0 | 0 | 1 | 1 | 0 | 0 | 0 | 0 | .00055 | 1.60944 | 0 | 0 | 0 | 0 | .00089 |
| agio | 2 | 0 | 1 | 0 | 2 | 5 | .00083 | 0 | .00101 | 0 | .00111 | .51083 | .00042 | 0 | .00051 | 0 | .00057 |
| agitazione | 0 | 0 | 1 | 0 | 0 | 1 | 0 | 0 | .00101 | 0 | 0 | 1.60944 | 0 | 0 | .00162 | 0 | 0 |
| alienante | 1 | 0 | 0 | 0 | 0 | 1 | .00041 | 0 | 0 | 0 | 0 | 1.60944 | .00066 | 0 | 0 | 0 | 0 |
| am* | 10 | 3 | 0 | 6 | 19 | 38 | .00413 | .00214 | 0 | .00336 | .01051 | .22314 | .00092 | .00048 | 0 | .00075 | .00235 |
| amareggiata | 0 | 0 | 0 | 0 | 1 | 1 | 0 | 0 | 0 | 0 | .00055 | 1.60944 | 0 | 0 | 0 | 0 | .00089 |
| amic* | 1 | 3 | 1 | 7 | 2 | 14 | .00041 | .00214 | .00101 | .00392 | .00111 | 0 | 0 | 0 | 0 | 0 | 0 |
| ammirata | 0 | 0 | 0 | 0 | 1 | 1 | 0 | 0 | 0 | 0 | .00055 | 1.60944 | 0 | 0 | 0 | 0 | .00089 |
| amorfo | 0 | 1 | 0 | 0 | 0 | 1 | 0 | .00071 | 0 | 0 | 0 | 1.60944 | 0 | .00115 | 0 | 0 | 0 |
| ampless* | 0 | 2 | 0 | 0 | 1 | 3 | 0 | .00142 | 0 | 0 | .00055 | .91629 | 0 | .00131 | 0 | 0 | .00051 |
| anaffettiva | 1 | 0 | 0 | 0 | 0 | 1 | .00041 | 0 | 0 | 0 | 0 | 1.60944 | .00066 | 0 | 0 | 0 | 0 |
| anale | 0 | 0 | 1 | 0 | 1 | 2 | 0 | 0 | .00101 | 0 | .00055 | .91629 | 0 | 0 | .00092 | 0 | .00051 |
| angosc* | 3 | 0 | 1 | 1 | 0 | 5 | .00124 | 0 | .00101 | .00056 | 0 | .51083 | .00063 | 0 | .00051 | .00029 | 0 |
| animalesc* | 1 | 1 | 0 | 0 | 0 | 2 | .00041 | .00071 | 0 | 0 | 0 | .91629 | .00038 | .00065 | 0 | 0 | 0 |
| annoi* | 3 | 2 | 1 | 0 | 1 | 7 | .00124 | .00142 | .00101 | 0 | .00055 | .22314 | .00028 | .00032 | .00022 | 0 | .00012 |
| anorgasmia | 1 | 0 | 0 | 0 | 0 | 1 | .00041 | 0 | 0 | 0 | 0 | 1.60944 | .00066 | 0 | 0 | 0 | 0 |
| ansi* | 22 | 6 | 3 | 0 | 3 | 34 | .00909 | .00427 | .00302 | 0 | .00166 | .22314 | .00203 | .00095 | .00067 | 0 | .00037 |
| anticoncezionale | 1 | 0 | 0 | 0 | 0 | 1 | .00041 | 0 | 0 | 0 | 0 | 1.60944 | .00066 | 0 | 0 | 0 | 0 |
| apati* | 3 | 2 | 0 | 0 | 2 | 7 | .00124 | .00142 | 0 | 0 | .00111 | .51083 | .00063 | .00073 | 0 | 0 | .00057 |
| app | 0 | 0 | 0 | 4 | 2 | 6 | 0 | 0 | 0 | .00224 | .00111 | .91629 | 0 | 0 | 0 | .00205 | .00101 |
| appag* | 15 | 5 | 6 | 0 | 9 | 35 | .00620 | .00356 | .00604 | 0 | .00498 | .22314 | .00138 | .00079 | .00135 | 0 | .00111 |
| appassion* | 2 | 0 | 0 | 0 | 1 | 3 | .00083 | 0 | 0 | 0 | .00055 | .91629 | .00076 | 0 | 0 | 0 | .00051 |
| appesantita | 1 | 0 | 0 | 0 | 0 | 1 | .00041 | 0 | 0 | 0 | 0 | 1.60944 | .00066 | 0 | 0 | 0 | 0 |
| appetito | 2 | 2 | 0 | 0 | 0 | 4 | .00083 | .00142 | 0 | 0 | 0 | .91629 | .00076 | .00131 | 0 | 0 | 0 |
| appiatt* | 1 | 2 | 0 | 0 | 0 | 3 | .00041 | .00142 | 0 | 0 | 0 | .91629 | .00038 | .00131 | 0 | 0 | 0 |
| apprezz* | 0 | 1 | 0 | 0 | 7 | 8 | 0 | .00071 | 0 | 0 | .00387 | .91629 | 0 | .00065 | 0 | 0 | .00355 |
| appuntament* | 1 | 0 | 2 | 0 | 0 | 3 | .00041 | 0 | .00201 | 0 | 0 | .91629 | .00038 | 0 | .00185 | 0 | 0 |
| arido | 1 | 0 | 0 | 0 | 0 | 1 | .00041 | 0 | 0 | 0 | 0 | 1.60944 | .00066 | 0 | 0 | 0 | 0 |
| armonia | 1 | 1 | 0 | 0 | 0 | 2 | .00041 | .00071 | 0 | 0 | 0 | .91629 | .00038 | .00065 | 0 | 0 | 0 |
| arrabbiat* | 2 | 0 | 0 | 0 | 0 | 2 | .00083 | 0 | 0 | 0 | 0 | 1.60944 | .00133 | 0 | 0 | 0 | 0 |
| arrend* | 2 | 0 | 0 | 0 | 0 | 2 | .00083 | 0 | 0 | 0 | 0 | 1.60944 | .00133 | 0 | 0 | 0 | 0 |
| asessual* | 4 | 0 | 0 | 0 | 0 | 4 | .00165 | 0 | 0 | 0 | 0 | 1.60944 | .00266 | 0 | 0 | 0 | 0 |
| asociale | 1 | 0 | 0 | 0 | 0 | 1 | .00041 | 0 | 0 | 0 | 0 | 1.60944 | .00066 | 0 | 0 | 0 | 0 |
| assaporato | 1 | 0 | 0 | 0 | 0 | 1 | .00041 | 0 | 0 | 0 | 0 | 1.60944 | .00066 | 0 | 0 | 0 | 0 |
| assecond* | 1 | 0 | 0 | 1 | 2 | 4 | .00041 | 0 | 0 | .00056 | .00111 | .51083 | .00021 | 0 | 0 | .00029 | .00057 |
| assemblamenti | 1 | 0 | 0 | 0 | 0 | 1 | .00041 | 0 | 0 | 0 | 0 | 1.60944 | .00066 | 0 | 0 | 0 | 0 |
| assillante | 1 | 0 | 0 | 0 | 0 | 1 | .00041 | 0 | 0 | 0 | 0 | 1.60944 | .00066 | 0 | 0 | 0 | 0 |
| assopi* | 2 | 2 | 0 | 0 | 1 | 5 | .00083 | .00142 | 0 | 0 | .00055 | .51083 | .00042 | .00073 | 0 | 0 | .00028 |
| assuefazione | 1 | 0 | 0 | 0 | 0 | 1 | .00041 | 0 | 0 | 0 | 0 | 1.60944 | .00066 | 0 | 0 | 0 | 0 |
| astenendomi | 0 | 0 | 0 | 0 | 1 | 1 | 0 | 0 | 0 | 0 | .00055 | 1.60944 | 0 | 0 | 0 | 0 | .00089 |
| astinenza | 7 | 3 | 0 | 0 | 3 | 13 | .00289 | .00214 | 0 | 0 | .00166 | .51083 | .00148 | .00109 | 0 | 0 | .00085 |
| attenzion* | 3 | 2 | 1 | 0 | 4 | 10 | .00124 | .00142 | .00101 | 0 | .00221 | .22314 | .00028 | .00032 | .00022 | 0 | .00049 |
| attività | 103 | 6 | 17 | 2 | 18 | 146 | .04254 | .00427 | .01712 | .00112 | .00996 | 0 | 0 | 0 | 0 | 0 | 0 |
| attra* | 0 | 2 | 0 | 1 | 7 | 10 | 0 | .00142 | 0 | .00056 | .00387 | .51083 | 0 | .00073 | 0 | .00029 | .00198 |
| attriti | 1 | 0 | 0 | 0 | 0 | 1 | .00041 | 0 | 0 | 0 | 0 | 1.60944 | .00066 | 0 | 0 | 0 | 0 |
| audio* | 0 | 0 | 0 | 2 | 0 | 2 | 0 | 0 | 0 | .00112 | 0 | 1.60944 | 0 | 0 | 0 | .00180 | 0 |
| ausil* | 0 | 0 | 0 | 71 | 2 | 73 | 0 | 0 | 0 | .03973 | .00111 | .91629 | 0 | 0 | 0 | .03641 | .00101 |
| autoeccitazione | 0 | 0 | 0 | 1 | 0 | 1 | 0 | 0 | 0 | .00056 | 0 | 1.60944 | 0 | 0 | 0 | .00090 | 0 |
| autoeroti* | 19 | 5 | 72 | 9 | 22 | 127 | .00785 | .00356 | .07251 | .00504 | .01217 | 0 | 0 | 0 | 0 | 0 | 0 |
| autostima | 0 | 0 | 0 | 0 | 1 | 1 | 0 | 0 | 0 | 0 | .00055 | 1.60944 | 0 | 0 | 0 | 0 | .00089 |
| avvicin* | 1 | 1 | 0 | 0 | 2 | 4 | .00041 | .00071 | 0 | 0 | .00111 | .51083 | .00021 | .00036 | 0 | 0 | .00057 |
| avvilente | 1 | 0 | 0 | 0 | 0 | 1 | .00041 | 0 | 0 | 0 | 0 | 1.60944 | .00066 | 0 | 0 | 0 | 0 |
| avvincente | 1 | 0 | 0 | 0 | 0 | 1 | .00041 | 0 | 0 | 0 | 0 | 1.60944 | .00066 | 0 | 0 | 0 | 0 |
| avvolgente | 1 | 0 | 0 | 0 | 0 | 1 | .00041 | 0 | 0 | 0 | 0 | 1.60944 | .00066 | 0 | 0 | 0 | 0 |
| bac* | 3 | 0 | 0 | 0 | 4 | 7 | .00124 | 0 | 0 | 0 | .00221 | .91629 | .00114 | 0 | 0 | 0 | .00203 |
| bang | 0 | 0 | 0 | 0 | 1 | 1 | 0 | 0 | 0 | 0 | .00055 | 1.60944 | 0 | 0 | 0 | 0 | .00089 |
| bdsm | 0 | 4 | 0 | 0 | 2 | 6 | 0 | .00285 | 0 | 0 | .00111 | .91629 | 0 | .00261 | 0 | 0 | .00101 |
| bellezza | 1 | 0 | 0 | 0 | 3 | 4 | .00041 | 0 | 0 | 0 | .00166 | .91629 | .00038 | 0 | 0 | 0 | .00152 |
| benessere | 1 | 0 | 1 | 0 | 2 | 4 | .00041 | 0 | .00101 | 0 | .00111 | .51083 | .00021 | 0 | .00051 | 0 | .00057 |
| biasimato | 0 | 0 | 0 | 0 | 1 | 1 | 0 | 0 | 0 | 0 | .00055 | 1.60944 | 0 | 0 | 0 | 0 | .00089 |
| bisessual* | 0 | 2 | 0 | 0 | 2 | 4 | 0 | .00142 | 0 | 0 | .00111 | .91629 | 0 | .00131 | 0 | 0 | .00101 |
| bisogn* | 9 | 7 | 20 | 11 | 24 | 71 | .00372 | .00499 | .02014 | .00616 | .01328 | 0 | 0 | 0 | 0 | 0 | 0 |
| blocc* | 5 | 0 | 1 | 0 | 3 | 9 | .00207 | 0 | .00101 | 0 | .00166 | .51083 | .00105 | 0 | .00051 | 0 | .00085 |
| bondage | 0 | 1 | 0 | 0 | 1 | 2 | 0 | .00071 | 0 | 0 | .00055 | .91629 | 0 | .00065 | 0 | 0 | .00051 |
| brio | 0 | 0 | 1 | 0 | 0 | 1 | 0 | 0 | .00101 | 0 | 0 | 1.60944 | 0 | 0 | .00162 | 0 | 0 |
| call | 1 | 0 | 0 | 0 | 0 | 1 | .00041 | 0 | 0 | 0 | 0 | 1.60944 | .00066 | 0 | 0 | 0 | 0 |
| calm* | 1 | 1 | 4 | 0 | 0 | 6 | .00041 | .00071 | .00403 | 0 | 0 | .51083 | .00021 | .00036 | .00206 | 0 | 0 |
| calor* | 1 | 0 | 0 | 0 | 1 | 2 | .00041 | 0 | 0 | 0 | .00055 | .91629 | .00038 | 0 | 0 | 0 | .00051 |
| canal* | 0 | 0 | 0 | 1 | 1 | 2 | 0 | 0 | 0 | .00056 | .00055 | .91629 | 0 | 0 | 0 | .00051 | .00051 |
| capelli | 0 | 0 | 0 | 0 | 1 | 1 | 0 | 0 | 0 | 0 | .00055 | 1.60944 | 0 | 0 | 0 | 0 | .00089 |
| capezzoli | 0 | 0 | 0 | 0 | 1 | 1 | 0 | 0 | 0 | 0 | .00055 | 1.60944 | 0 | 0 | 0 | 0 | .00089 |
| cardiaca | 0 | 0 | 0 | 0 | 1 | 1 | 0 | 0 | 0 | 0 | .00055 | 1.60944 | 0 | 0 | 0 | 0 | .00089 |
| carezz* | 2 | 0 | 0 | 0 | 3 | 5 | .00083 | 0 | 0 | 0 | .00166 | .91629 | .00076 | 0 | 0 | 0 | .00152 |
| carn* | 5 | 0 | 0 | 0 | 2 | 7 | .00207 | 0 | 0 | 0 | .00111 | .91629 | .00189 | 0 | 0 | 0 | .00101 |
| cartone | 0 | 0 | 0 | 1 | 0 | 1 | 0 | 0 | 0 | .00056 | 0 | 1.60944 | 0 | 0 | 0 | .00090 | 0 |
| castità | 0 | 1 | 0 | 0 | 0 | 1 | 0 | .00071 | 0 | 0 | 0 | 1.60944 | 0 | .00115 | 0 | 0 | 0 |
| castrazione | 0 | 1 | 0 | 0 | 0 | 1 | 0 | .00071 | 0 | 0 | 0 | 1.60944 | 0 | .00115 | 0 | 0 | 0 |
| cellular* | 0 | 0 | 0 | 3 | 0 | 3 | 0 | 0 | 0 | .00168 | 0 | 1.60944 | 0 | 0 | 0 | .00270 | 0 |
| cervice | 0 | 0 | 0 | 0 | 1 | 1 | 0 | 0 | 0 | 0 | .00055 | 1.60944 | 0 | 0 | 0 | 0 | .00089 |
| chat | 0 | 2 | 2 | 12 | 1 | 17 | 0 | .00142 | .00201 | .00672 | .00055 | .22314 | 0 | .00032 | .00045 | .00150 | .00012 |
| chiam* | 0 | 1 | 1 | 5 | 1 | 8 | 0 | .00071 | .00101 | .00280 | .00055 | .22314 | 0 | .00016 | .00022 | .00062 | .00012 |
| cicl* | 3 | 1 | 0 | 0 | 0 | 4 | .00124 | .00071 | 0 | 0 | 0 | .91629 | .00114 | .00065 | 0 | 0 | 0 |
| cistite | 1 | 0 | 0 | 0 | 0 | 1 | .00041 | 0 | 0 | 0 | 0 | 1.60944 | .00066 | 0 | 0 | 0 | 0 |
| clandestina | 0 | 0 | 0 | 0 | 1 | 1 | 0 | 0 | 0 | 0 | .00055 | 1.60944 | 0 | 0 | 0 | 0 | .00089 |
| clitoride* | 0 | 0 | 1 | 2 | 0 | 3 | 0 | 0 | .00101 | .00112 | 0 | .91629 | 0 | 0 | .00092 | .00103 | 0 |
| coccol* | 1 | 1 | 0 | 0 | 2 | 4 | .00041 | .00071 | 0 | 0 | .00111 | .51083 | .00021 | .00036 | 0 | 0 | .00057 |
| cock | 0 | 0 | 0 | 1 | 0 | 1 | 0 | 0 | 0 | .00056 | 0 | 1.60944 | 0 | 0 | 0 | .00090 | 0 |
| coetanee | 0 | 0 | 1 | 0 | 0 | 1 | 0 | 0 | .00101 | 0 | 0 | 1.60944 | 0 | 0 | .00162 | 0 | 0 |
| coinquilin* | 1 | 0 | 0 | 1 | 1 | 3 | .00041 | 0 | 0 | .00056 | .00055 | .51083 | .00021 | 0 | 0 | .00029 | .00028 |
| coinvol* | 10 | 2 | 5 | 1 | 4 | 22 | .00413 | .00142 | .00504 | .00056 | .00221 | 0 | 0 | 0 | 0 | 0 | 0 |
| coitale | 1 | 0 | 0 | 0 | 0 | 1 | .00041 | 0 | 0 | 0 | 0 | 1.60944 | .00066 | 0 | 0 | 0 | 0 |
| colp* | 2 | 0 | 1 | 1 | 2 | 6 | .00083 | 0 | .00101 | .00056 | .00111 | .22314 | .00018 | 0 | .00022 | .00012 | .00025 |
| coltiva* | 2 | 0 | 0 | 0 | 1 | 3 | .00083 | 0 | 0 | 0 | .00055 | .91629 | .00076 | 0 | 0 | 0 | .00051 |
| comic* | 2 | 0 | 0 | 0 | 0 | 2 | .00083 | 0 | 0 | 0 | 0 | 1.60944 | .00133 | 0 | 0 | 0 | 0 |
| comod* | 3 | 0 | 0 | 0 | 0 | 3 | .00124 | 0 | 0 | 0 | 0 | 1.60944 | .00199 | 0 | 0 | 0 | 0 |
| compagn* | 19 | 10 | 7 | 12 | 7 | 55 | .00785 | .00712 | .00705 | .00672 | .00387 | 0 | 0 | 0 | 0 | 0 | 0 |
| compens* | 3 | 1 | 0 | 1 | 0 | 5 | .00124 | .00071 | 0 | .00056 | 0 | .51083 | .00063 | .00036 | 0 | .00029 | 0 |
| complic* | 3 | 1 | 2 | 0 | 4 | 10 | .00124 | .00071 | .00201 | 0 | .00221 | .22314 | .00028 | .00016 | .00045 | 0 | .00049 |
| comprensione | 1 | 1 | 0 | 0 | 1 | 3 | .00041 | .00071 | 0 | 0 | .00055 | .51083 | .00021 | .00036 | 0 | 0 | .00028 |
| compromesso | 1 | 0 | 0 | 0 | 0 | 1 | .00041 | 0 | 0 | 0 | 0 | 1.60944 | .00066 | 0 | 0 | 0 | 0 |
| computer | 0 | 0 | 0 | 1 | 0 | 1 | 0 | 0 | 0 | .00056 | 0 | 1.60944 | 0 | 0 | 0 | .00090 | 0 |
| comunic* | 3 | 1 | 0 | 2 | 4 | 10 | .00124 | .00071 | 0 | .00112 | .00221 | .22314 | .00028 | .00016 | 0 | .00025 | .00049 |
| conceder* | 0 | 0 | 0 | 0 | 2 | 2 | 0 | 0 | 0 | 0 | .00111 | 1.60944 | 0 | 0 | 0 | 0 | .00178 |
| concess* | 1 | 0 | 0 | 0 | 2 | 3 | .00041 | 0 | 0 | 0 | .00111 | .91629 | .00038 | 0 | 0 | 0 | .00101 |
| concezionale | 0 | 1 | 0 | 0 | 0 | 1 | 0 | .00071 | 0 | 0 | 0 | 1.60944 | 0 | .00115 | 0 | 0 | 0 |
| condivi* | 7 | 4 | 9 | 5 | 15 | 40 | .00289 | .00285 | .00906 | .00280 | .00830 | 0 | 0 | 0 | 0 | 0 | 0 |
| confidenza | 0 | 0 | 0 | 1 | 0 | 1 | 0 | 0 | 0 | .00056 | 0 | 1.60944 | 0 | 0 | 0 | .00090 | 0 |
| conflitt* | 2 | 0 | 1 | 0 | 0 | 3 | .00083 | 0 | .00101 | 0 | 0 | .91629 | .00076 | 0 | .00092 | 0 | 0 |
| confortante | 1 | 0 | 0 | 0 | 0 | 1 | .00041 | 0 | 0 | 0 | 0 | 1.60944 | .00066 | 0 | 0 | 0 | 0 |
| confront* | 0 | 0 | 0 | 1 | 1 | 2 | 0 | 0 | 0 | .00056 | .00055 | .91629 | 0 | 0 | 0 | .00051 | .00051 |
| confuso | 3 | 0 | 0 | 0 | 0 | 3 | .00124 | 0 | 0 | 0 | 0 | 1.60944 | .00199 | 0 | 0 | 0 | 0 |
| congiunt* | 2 | 0 | 1 | 1 | 0 | 4 | .00083 | 0 | .00101 | .00056 | 0 | .51083 | .00042 | 0 | .00051 | .00029 | 0 |
| coniuge | 0 | 1 | 0 | 0 | 0 | 1 | 0 | .00071 | 0 | 0 | 0 | 1.60944 | 0 | .00115 | 0 | 0 | 0 |
| conosc* | 3 | 0 | 0 | 2 | 12 | 17 | .00124 | 0 | 0 | .00112 | .00664 | .51083 | .00063 | 0 | 0 | .00057 | .00339 |
| conquista | 0 | 0 | 0 | 0 | 1 | 1 | 0 | 0 | 0 | 0 | .00055 | 1.60944 | 0 | 0 | 0 | 0 | .00089 |
| consapevol* | 13 | 1 | 3 | 0 | 37 | 54 | .00537 | .00071 | .00302 | 0 | .02048 | .22314 | .00120 | .00016 | .00067 | 0 | .00457 |
| consolatorio | 1 | 0 | 0 | 0 | 0 | 1 | .00041 | 0 | 0 | 0 | 0 | 1.60944 | .00066 | 0 | 0 | 0 | 0 |
| consumazione | 1 | 0 | 0 | 0 | 0 | 1 | .00041 | 0 | 0 | 0 | 0 | 1.60944 | .00066 | 0 | 0 | 0 | 0 |
| contatt* | 31 | 6 | 3 | 2 | 30 | 72 | .01280 | .00427 | .00302 | .00112 | .01660 | 0 | 0 | 0 | 0 | 0 | 0 |
| contenta | 0 | 0 | 0 | 0 | 1 | 1 | 0 | 0 | 0 | 0 | .00055 | 1.60944 | 0 | 0 | 0 | 0 | .00089 |
| contrastante | 2 | 0 | 0 | 0 | 0 | 2 | .00083 | 0 | 0 | 0 | 0 | 1.60944 | .00133 | 0 | 0 | 0 | 0 |
| controvoglia | 1 | 0 | 0 | 0 | 0 | 1 | .00041 | 0 | 0 | 0 | 0 | 1.60944 | .00066 | 0 | 0 | 0 | 0 |
| conversazion* | 0 | 1 | 1 | 2 | 2 | 6 | 0 | .00071 | .00101 | .00112 | .00111 | .22314 | 0 | .00016 | .00022 | .00025 | .00025 |
| convi* | 15 | 1 | 3 | 3 | 1 | 23 | .00620 | .00071 | .00302 | .00168 | .00055 | 0 | 0 | 0 | 0 | 0 | 0 |
| coppia | 18 | 1 | 3 | 8 | 15 | 45 | .00743 | .00071 | .00302 | .00448 | .00830 | 0 | 0 | 0 | 0 | 0 | 0 |
| corp* | 19 | 2 | 9 | 3 | 72 | 105 | .00785 | .00142 | .00906 | .00168 | .03985 | 0 | 0 | 0 | 0 | 0 | 0 |
| creative | 0 | 1 | 0 | 0 | 0 | 1 | 0 | .00071 | 0 | 0 | 0 | 1.60944 | 0 | .00115 | 0 | 0 | 0 |
| crudo | 0 | 1 | 0 | 0 | 0 | 1 | 0 | .00071 | 0 | 0 | 0 | 1.60944 | 0 | .00115 | 0 | 0 | 0 |
| culmine | 0 | 0 | 0 | 0 | 1 | 1 | 0 | 0 | 0 | 0 | .00055 | 1.60944 | 0 | 0 | 0 | 0 | .00089 |
| cura | 0 | 0 | 1 | 0 | 2 | 3 | 0 | 0 | .00101 | 0 | .00111 | .91629 | 0 | 0 | .00092 | 0 | .00101 |
| curios* | 7 | 2 | 1 | 1 | 3 | 14 | .00289 | .00142 | .00101 | .00056 | .00166 | 0 | 0 | 0 | 0 | 0 | 0 |
| dating | 0 | 0 | 0 | 1 | 0 | 1 | 0 | 0 | 0 | .00056 | 0 | 1.60944 | 0 | 0 | 0 | .00090 | 0 |
| debol* | 1 | 0 | 0 | 0 | 1 | 2 | .00041 | 0 | 0 | 0 | .00055 | .91629 | .00038 | 0 | 0 | 0 | .00051 |
| dedic* | 10 | 2 | 3 | 1 | 8 | 24 | .00413 | .00142 | .00302 | .00056 | .00443 | 0 | 0 | 0 | 0 | 0 | 0 |
| delicata | 0 | 0 | 1 | 0 | 1 | 2 | 0 | 0 | .00101 | 0 | .00055 | .91629 | 0 | 0 | .00092 | 0 | .00051 |
| delud* | 2 | 0 | 0 | 0 | 1 | 3 | .00083 | 0 | 0 | 0 | .00055 | .91629 | .00076 | 0 | 0 | 0 | .00051 |
| depr* | 8 | 1 | 0 | 0 | 0 | 9 | .00330 | .00071 | 0 | 0 | 0 | .91629 | .00303 | .00065 | 0 | 0 | 0 |
| desider* | 119 | 215 | 29 | 11 | 41 | 415 | .04915 | .15313 | .02920 | .00616 | .02269 | 0 | 0 | 0 | 0 | 0 | 0 |
| destabilizzata | 1 | 0 | 0 | 0 | 0 | 1 | .00041 | 0 | 0 | 0 | 0 | 1.60944 | .00066 | 0 | 0 | 0 | 0 |
| detesto | 0 | 0 | 0 | 0 | 1 | 1 | 0 | 0 | 0 | 0 | .00055 | 1.60944 | 0 | 0 | 0 | 0 | .00089 |
| deumanizzanti | 0 | 0 | 0 | 0 | 1 | 1 | 0 | 0 | 0 | 0 | .00055 | 1.60944 | 0 | 0 | 0 | 0 | .00089 |
| dialog* | 3 | 0 | 0 | 0 | 3 | 6 | .00124 | 0 | 0 | 0 | .00166 | .91629 | .00114 | 0 | 0 | 0 | .00152 |
| difett* | 0 | 0 | 0 | 0 | 3 | 3 | 0 | 0 | 0 | 0 | .00166 | 1.60944 | 0 | 0 | 0 | 0 | .00267 |
| digitali | 1 | 1 | 0 | 0 | 0 | 2 | .00041 | .00071 | 0 | 0 | 0 | .91629 | .00038 | .00065 | 0 | 0 | 0 |
| digiuno | 0 | 0 | 0 | 0 | 1 | 1 | 0 | 0 | 0 | 0 | .00055 | 1.60944 | 0 | 0 | 0 | 0 | .00089 |
| dilatazione | 1 | 0 | 0 | 1 | 0 | 2 | .00041 | 0 | 0 | .00056 | 0 | .91629 | .00038 | 0 | 0 | .00051 | 0 |
| dildo | 0 | 1 | 0 | 1 | 0 | 2 | 0 | .00071 | 0 | .00056 | 0 | .91629 | 0 | .00065 | 0 | .00051 | 0 |
| dipend* | 0 | 0 | 0 | 0 | 4 | 4 | 0 | 0 | 0 | 0 | .00221 | 1.60944 | 0 | 0 | 0 | 0 | .00356 |
| diplomatica | 1 | 0 | 0 | 0 | 0 | 1 | .00041 | 0 | 0 | 0 | 0 | 1.60944 | .00066 | 0 | 0 | 0 | 0 |
| dirty | 1 | 0 | 0 | 1 | 0 | 2 | .00041 | 0 | 0 | .00056 | 0 | .91629 | .00038 | 0 | 0 | .00051 | 0 |
| disagio | 3 | 1 | 0 | 1 | 2 | 7 | .00124 | .00071 | 0 | .00056 | .00111 | .22314 | .00028 | .00016 | 0 | .00012 | .00025 |
| discors* | 1 | 0 | 0 | 1 | 0 | 2 | .00041 | 0 | 0 | .00056 | 0 | .91629 | .00038 | 0 | 0 | .00051 | 0 |
| disgustato | 0 | 0 | 0 | 0 | 1 | 1 | 0 | 0 | 0 | 0 | .00055 | 1.60944 | 0 | 0 | 0 | 0 | .00089 |
| disinibit* | 4 | 1 | 0 | 0 | 1 | 6 | .00165 | .00071 | 0 | 0 | .00055 | .51083 | .00084 | .00036 | 0 | 0 | .00028 |
| disinteress* | 2 | 0 | 2 | 0 | 1 | 5 | .00083 | 0 | .00201 | 0 | .00055 | .51083 | .00042 | 0 | .00103 | 0 | .00028 |
| disperata | 1 | 0 | 0 | 0 | 0 | 1 | .00041 | 0 | 0 | 0 | 0 | 1.60944 | .00066 | 0 | 0 | 0 | 0 |
| dispiac* | 3 | 0 | 0 | 0 | 2 | 5 | .00124 | 0 | 0 | 0 | .00111 | .91629 | .00114 | 0 | 0 | 0 | .00101 |
| disponibil* | 3 | 2 | 1 | 0 | 1 | 7 | .00124 | .00142 | .00101 | 0 | .00055 | .22314 | .00028 | .00032 | .00022 | 0 | .00012 |
| dispositivi | 0 | 0 | 0 | 1 | 0 | 1 | 0 | 0 | 0 | .00056 | 0 | 1.60944 | 0 | 0 | 0 | .00090 | 0 |
| distacc* | 3 | 0 | 0 | 0 | 2 | 5 | .00124 | 0 | 0 | 0 | .00111 | .91629 | .00114 | 0 | 0 | 0 | .00101 |
| distan* | 26 | 8 | 5 | 3 | 6 | 48 | .01074 | .00570 | .00504 | .00168 | .00332 | 0 | 0 | 0 | 0 | 0 | 0 |
| distancing | 1 | 0 | 0 | 0 | 0 | 1 | .00041 | 0 | 0 | 0 | 0 | 1.60944 | .00066 | 0 | 0 | 0 | 0 |
| dita | 0 | 0 | 0 | 1 | 0 | 1 | 0 | 0 | 0 | .00056 | 0 | 1.60944 | 0 | 0 | 0 | .00090 | 0 |
| divert* | 10 | 1 | 4 | 0 | 2 | 17 | .00413 | .00071 | .00403 | 0 | .00111 | .22314 | .00092 | .00016 | .00090 | 0 | .00025 |
| doccia | 0 | 1 | 3 | 0 | 0 | 4 | 0 | .00071 | .00302 | 0 | 0 | .91629 | 0 | .00065 | .00277 | 0 | 0 |
| dolor* | 3 | 1 | 0 | 0 | 0 | 4 | .00124 | .00071 | 0 | 0 | 0 | .91629 | .00114 | .00065 | 0 | 0 | 0 |
| domin* | 0 | 1 | 0 | 1 | 0 | 2 | 0 | .00071 | 0 | .00056 | 0 | .91629 | 0 | .00065 | 0 | .00051 | 0 |
| durex | 0 | 0 | 0 | 1 | 0 | 1 | 0 | 0 | 0 | .00056 | 0 | 1.60944 | 0 | 0 | 0 | .00090 | 0 |
| eccit* | 26 | 111 | 8 | 12 | 13 | 170 | .01074 | .07906 | .00806 | .00672 | .00719 | 0 | 0 | 0 | 0 | 0 | 0 |
| edonistico | 0 | 1 | 0 | 0 | 0 | 1 | 0 | .00071 | 0 | 0 | 0 | 1.60944 | 0 | .00115 | 0 | 0 | 0 |
| effusioni | 1 | 0 | 0 | 0 | 0 | 1 | .00041 | 0 | 0 | 0 | 0 | 1.60944 | .00066 | 0 | 0 | 0 | 0 |
| egocentrica | 1 | 0 | 0 | 0 | 0 | 1 | .00041 | 0 | 0 | 0 | 0 | 1.60944 | .00066 | 0 | 0 | 0 | 0 |
| elicitata | 0 | 0 | 1 | 0 | 0 | 1 | 0 | 0 | .00101 | 0 | 0 | 1.60944 | 0 | 0 | .00162 | 0 | 0 |
| emancipazione | 1 | 0 | 0 | 0 | 0 | 1 | .00041 | 0 | 0 | 0 | 0 | 1.60944 | .00066 | 0 | 0 | 0 | 0 |
| emollienti | 0 | 0 | 0 | 1 | 0 | 1 | 0 | 0 | 0 | .00056 | 0 | 1.60944 | 0 | 0 | 0 | .00090 | 0 |
| emotiv* | 3 | 1 | 2 | 0 | 5 | 11 | .00124 | .00071 | .00201 | 0 | .00277 | .22314 | .00028 | .00016 | .00045 | 0 | .00062 |
| emozion* | 1 | 0 | 0 | 0 | 6 | 7 | .00041 | 0 | 0 | 0 | .00332 | .91629 | .00038 | 0 | 0 | 0 | .00304 |
| empatia | 0 | 1 | 0 | 0 | 0 | 1 | 0 | .00071 | 0 | 0 | 0 | 1.60944 | 0 | .00115 | 0 | 0 | 0 |
| energ* | 6 | 0 | 1 | 0 | 3 | 10 | .00248 | 0 | .00101 | 0 | .00166 | .51083 | .00127 | 0 | .00051 | 0 | .00085 |
| entusiasm* | 1 | 0 | 1 | 0 | 1 | 3 | .00041 | 0 | .00101 | 0 | .00055 | .51083 | .00021 | 0 | .00051 | 0 | .00028 |
| equilibr* | 2 | 1 | 1 | 0 | 2 | 6 | .00083 | .00071 | .00101 | 0 | .00111 | .22314 | .00018 | .00016 | .00022 | 0 | .00025 |
| erba | 0 | 0 | 0 | 0 | 1 | 1 | 0 | 0 | 0 | 0 | .00055 | 1.60944 | 0 | 0 | 0 | 0 | .00089 |
| erezione | 0 | 0 | 1 | 0 | 0 | 1 | 0 | 0 | .00101 | 0 | 0 | 1.60944 | 0 | 0 | .00162 | 0 | 0 |
| erogen* | 0 | 2 | 0 | 0 | 2 | 4 | 0 | .00142 | 0 | 0 | .00111 | .91629 | 0 | .00131 | 0 | 0 | .00101 |
| erot* | 11 | 109 | 5 | 33 | 4 | 162 | .00454 | .07764 | .00504 | .01847 | .00221 | 0 | 0 | 0 | 0 | 0 | 0 |
| esasperante | 1 | 0 | 0 | 0 | 0 | 1 | .00041 | 0 | 0 | 0 | 0 | 1.60944 | .00066 | 0 | 0 | 0 | 0 |
| esauri* | 0 | 1 | 1 | 0 | 0 | 2 | 0 | .00071 | .00101 | 0 | 0 | .91629 | 0 | .00065 | .00092 | 0 | 0 |
| esigenz* | 3 | 1 | 1 | 1 | 4 | 10 | .00124 | .00071 | .00101 | .00056 | .00221 | 0 | 0 | 0 | 0 | 0 | 0 |
| esperienz* | 3 | 0 | 7 | 30 | 19 | 59 | .00124 | 0 | .00705 | .01679 | .01051 | .22314 | .00028 | 0 | .00157 | .00375 | .00235 |
| esperimento | 0 | 0 | 0 | 1 | 0 | 1 | 0 | 0 | 0 | .00056 | 0 | 1.60944 | 0 | 0 | 0 | .00090 | 0 |
| esplicit* | 0 | 1 | 1 | 76 | 4 | 82 | 0 | .00071 | .00101 | .04253 | .00221 | .22314 | 0 | .00016 | .00022 | .00949 | .00049 |
| esplodo | 1 | 0 | 0 | 0 | 0 | 1 | .00041 | 0 | 0 | 0 | 0 | 1.60944 | .00066 | 0 | 0 | 0 | 0 |
| esplor* | 5 | 2 | 2 | 1 | 6 | 16 | .00207 | .00142 | .00201 | .00056 | .00332 | 0 | 0 | 0 | 0 | 0 | 0 |
| espr* | 3 | 6 | 0 | 0 | 2 | 11 | .00124 | .00427 | 0 | 0 | .00111 | .51083 | .00063 | .00218 | 0 | 0 | .00057 |
| estraneazione | 1 | 0 | 0 | 0 | 0 | 1 | .00041 | 0 | 0 | 0 | 0 | 1.60944 | .00066 | 0 | 0 | 0 | 0 |
| estraniarmi | 1 | 0 | 0 | 0 | 0 | 1 | .00041 | 0 | 0 | 0 | 0 | 1.60944 | .00066 | 0 | 0 | 0 | 0 |
| estrem* | 0 | 3 | 0 | 0 | 2 | 5 | 0 | .00214 | 0 | 0 | .00111 | .91629 | 0 | .00196 | 0 | 0 | .00101 |
| eterosessual* | 0 | 1 | 0 | 0 | 1 | 2 | 0 | .00071 | 0 | 0 | .00055 | .91629 | 0 | .00065 | 0 | 0 | .00051 |
| facebook | 1 | 0 | 0 | 0 | 0 | 1 | .00041 | 0 | 0 | 0 | 0 | 1.60944 | .00066 | 0 | 0 | 0 | 0 |
| falli | 0 | 0 | 0 | 1 | 0 | 1 | 0 | 0 | 0 | .00056 | 0 | 1.60944 | 0 | 0 | 0 | .00090 | 0 |
| fallimento | 1 | 0 | 0 | 0 | 0 | 1 | .00041 | 0 | 0 | 0 | 0 | 1.60944 | .00066 | 0 | 0 | 0 | 0 |
| famiglia | 5 | 1 | 2 | 0 | 0 | 8 | .00207 | .00071 | .00201 | 0 | 0 | .51083 | .00105 | .00036 | .00103 | 0 | 0 |
| familiar* | 3 | 0 | 1 | 0 | 0 | 4 | .00124 | 0 | .00101 | 0 | 0 | .91629 | .00114 | 0 | .00092 | 0 | 0 |
| fantas* | 73 | 260 | 17 | 21 | 27 | 398 | .03015 | .18519 | .01712 | .01175 | .01494 | 0 | 0 | 0 | 0 | 0 | 0 |
| fastidio* | 2 | 1 | 0 | 0 | 2 | 5 | .00083 | .00071 | 0 | 0 | .00111 | .51083 | .00042 | .00036 | 0 | 0 | .00057 |
| fatic* | 5 | 2 | 5 | 0 | 0 | 12 | .00207 | .00142 | .00504 | 0 | 0 | .51083 | .00105 | .00073 | .00257 | 0 | 0 |
| felic* | 5 | 0 | 0 | 0 | 2 | 7 | .00207 | 0 | 0 | 0 | .00111 | .91629 | .00189 | 0 | 0 | 0 | .00101 |
| femminil* | 1 | 1 | 0 | 0 | 1 | 3 | .00041 | .00071 | 0 | 0 | .00055 | .51083 | .00021 | .00036 | 0 | 0 | .00028 |
| feticci | 0 | 0 | 0 | 0 | 1 | 1 | 0 | 0 | 0 | 0 | .00055 | 1.60944 | 0 | 0 | 0 | 0 | .00089 |
| fid* | 3 | 0 | 0 | 0 | 5 | 8 | .00124 | 0 | 0 | 0 | .00277 | .91629 | .00114 | 0 | 0 | 0 | .00254 |
| fidanzat* | 8 | 4 | 6 | 3 | 0 | 21 | .00330 | .00285 | .00604 | .00168 | 0 | .22314 | .00074 | .00064 | .00135 | .00037 | 0 |
| figa | 0 | 0 | 1 | 0 | 0 | 1 | 0 | 0 | .00101 | 0 | 0 | 1.60944 | 0 | 0 | .00162 | 0 | 0 |
| figl* | 4 | 0 | 0 | 0 | 3 | 7 | .00165 | 0 | 0 | 0 | .00166 | .91629 | .00151 | 0 | 0 | 0 | .00152 |
| film* | 0 | 0 | 2 | 7 | 0 | 9 | 0 | 0 | .00201 | .00392 | 0 | .91629 | 0 | 0 | .00185 | .00359 | 0 |
| fingere | 0 | 0 | 0 | 0 | 1 | 1 | 0 | 0 | 0 | 0 | .00055 | 1.60944 | 0 | 0 | 0 | 0 | .00089 |
| finta | 0 | 0 | 0 | 0 | 1 | 1 | 0 | 0 | 0 | 0 | .00055 | 1.60944 | 0 | 0 | 0 | 0 | .00089 |
| fisic* | 33 | 9 | 3 | 4 | 34 | 83 | .01363 | .00641 | .00302 | .00224 | .01882 | 0 | 0 | 0 | 0 | 0 | 0 |
| fisiologic* | 3 | 1 | 2 | 0 | 0 | 6 | .00124 | .00071 | .00201 | 0 | 0 | .51083 | .00063 | .00036 | .00103 | 0 | 0 |
| fiss* | 1 | 0 | 0 | 1 | 0 | 2 | .00041 | 0 | 0 | .00056 | 0 | .91629 | .00038 | 0 | 0 | .00051 | 0 |
| fittizia | 0 | 1 | 0 | 0 | 0 | 1 | 0 | .00071 | 0 | 0 | 0 | 1.60944 | 0 | .00115 | 0 | 0 | 0 |
| flirt | 0 | 0 | 0 | 1 | 1 | 2 | 0 | 0 | 0 | .00056 | .00055 | .91629 | 0 | 0 | 0 | .00051 | .00051 |
| fluid* | 1 | 0 | 1 | 0 | 0 | 2 | .00041 | 0 | .00101 | 0 | 0 | .91629 | .00038 | 0 | .00092 | 0 | 0 |
| foga | 0 | 0 | 0 | 0 | 1 | 1 | 0 | 0 | 0 | 0 | .00055 | 1.60944 | 0 | 0 | 0 | 0 | .00089 |
| forzat* | 7 | 1 | 0 | 1 | 2 | 11 | .00289 | .00071 | 0 | .00056 | .00111 | .22314 | .00065 | .00016 | 0 | .00012 | .00025 |
| foto* | 4 | 0 | 3 | 27 | 2 | 36 | .00165 | 0 | .00302 | .01511 | .00111 | .22314 | .00037 | 0 | .00067 | .00337 | .00025 |
| fratello | 1 | 0 | 1 | 0 | 0 | 2 | .00041 | 0 | .00101 | 0 | 0 | .91629 | .00038 | 0 | .00092 | 0 | 0 |
| fredd* | 1 | 0 | 0 | 1 | 1 | 3 | .00041 | 0 | 0 | .00056 | .00055 | .51083 | .00021 | 0 | 0 | .00029 | .00028 |
| frenata | 0 | 0 | 0 | 0 | 1 | 1 | 0 | 0 | 0 | 0 | .00055 | 1.60944 | 0 | 0 | 0 | 0 | .00089 |
| frenesi* | 1 | 0 | 0 | 0 | 2 | 3 | .00041 | 0 | 0 | 0 | .00111 | .91629 | .00038 | 0 | 0 | 0 | .00101 |
| frenetic* | 2 | 0 | 0 | 0 | 3 | 5 | .00083 | 0 | 0 | 0 | .00166 | .91629 | .00076 | 0 | 0 | 0 | .00152 |
| frequen* | 37 | 13 | 44 | 8 | 9 | 111 | .01528 | .00926 | .04431 | .00448 | .00498 | 0 | 0 | 0 | 0 | 0 | 0 |
| frett* | 6 | 1 | 0 | 0 | 2 | 9 | .00248 | .00071 | 0 | 0 | .00111 | .51083 | .00127 | .00036 | 0 | 0 | .00057 |
| frustr* | 20 | 4 | 3 | 0 | 3 | 30 | .00826 | .00285 | .00302 | 0 | .00166 | .22314 | .00184 | .00064 | .00067 | 0 | .00037 |
| fugace | 0 | 0 | 0 | 0 | 1 | 1 | 0 | 0 | 0 | 0 | .00055 | 1.60944 | 0 | 0 | 0 | 0 | .00089 |
| fuggente | 1 | 0 | 0 | 0 | 0 | 1 | .00041 | 0 | 0 | 0 | 0 | 1.60944 | .00066 | 0 | 0 | 0 | 0 |
| fumetti | 0 | 0 | 0 | 1 | 0 | 1 | 0 | 0 | 0 | .00056 | 0 | 1.60944 | 0 | 0 | 0 | .00090 | 0 |
| furiosa | 0 | 0 | 0 | 0 | 1 | 1 | 0 | 0 | 0 | 0 | .00055 | 1.60944 | 0 | 0 | 0 | 0 | .00089 |
| furtiv* | 1 | 1 | 0 | 0 | 0 | 2 | .00041 | .00071 | 0 | 0 | 0 | .91629 | .00038 | .00065 | 0 | 0 | 0 |
| gadget | 0 | 0 | 0 | 1 | 0 | 1 | 0 | 0 | 0 | .00056 | 0 | 1.60944 | 0 | 0 | 0 | .00090 | 0 |
| gang | 0 | 0 | 0 | 0 | 1 | 1 | 0 | 0 | 0 | 0 | .00055 | 1.60944 | 0 | 0 | 0 | 0 | .00089 |
| gender | 0 | 0 | 0 | 0 | 1 | 1 | 0 | 0 | 0 | 0 | .00055 | 1.60944 | 0 | 0 | 0 | 0 | .00089 |
| genere | 3 | 4 | 1 | 0 | 5 | 13 | .00124 | .00285 | .00101 | 0 | .00277 | .22314 | .00028 | .00064 | .00022 | 0 | .00062 |
| genital* | 1 | 0 | 0 | 0 | 1 | 2 | .00041 | 0 | 0 | 0 | .00055 | .91629 | .00038 | 0 | 0 | 0 | .00051 |
| genitor* | 5 | 1 | 5 | 2 | 1 | 14 | .00207 | .00071 | .00504 | .00112 | .00055 | 0 | 0 | 0 | 0 | 0 | 0 |
| gioc* | 8 | 1 | 6 | 10 | 5 | 30 | .00330 | .00071 | .00604 | .00560 | .00277 | 0 | 0 | 0 | 0 | 0 | 0 |
| giornalier* | 2 | 0 | 2 | 0 | 1 | 5 | .00083 | 0 | .00201 | 0 | .00055 | .51083 | .00042 | 0 | .00103 | 0 | .00028 |
| giud* | 1 | 0 | 0 | 0 | 2 | 3 | .00041 | 0 | 0 | 0 | .00111 | .91629 | .00038 | 0 | 0 | 0 | .00101 |
| giustific* | 2 | 0 | 0 | 0 | 1 | 3 | .00083 | 0 | 0 | 0 | .00055 | .91629 | .00076 | 0 | 0 | 0 | .00051 |
| god* | 5 | 0 | 1 | 1 | 4 | 11 | .00207 | 0 | .00101 | .00056 | .00221 | .22314 | .00046 | 0 | .00022 | .00012 | .00049 |
| gradi* | 0 | 2 | 1 | 1 | 0 | 4 | 0 | .00142 | .00101 | .00056 | 0 | .51083 | 0 | .00073 | .00051 | .00029 | 0 |
| gratific* | 2 | 1 | 1 | 0 | 0 | 4 | .00083 | .00071 | .00101 | 0 | 0 | .51083 | .00042 | .00036 | .00051 | 0 | 0 |
| guant* | 0 | 0 | 0 | 1 | 1 | 2 | 0 | 0 | 0 | .00056 | .00055 | .91629 | 0 | 0 | 0 | .00051 | .00051 |
| guard* | 6 | 0 | 4 | 11 | 8 | 29 | .00248 | 0 | .00403 | .00616 | .00443 | .22314 | .00055 | 0 | .00090 | .00137 | .00099 |
| gust* | 0 | 0 | 2 | 1 | 0 | 3 | 0 | 0 | .00201 | .00056 | 0 | .91629 | 0 | 0 | .00185 | .00051 | 0 |
| hard | 0 | 0 | 1 | 1 | 0 | 2 | 0 | 0 | .00101 | .00056 | 0 | .91629 | 0 | 0 | .00092 | .00051 | 0 |
| harem | 0 | 1 | 0 | 0 | 0 | 1 | 0 | .00071 | 0 | 0 | 0 | 1.60944 | 0 | .00115 | 0 | 0 | 0 |
| hot | 0 | 0 | 1 | 1 | 0 | 2 | 0 | 0 | .00101 | .00056 | 0 | .91629 | 0 | 0 | .00092 | .00051 | 0 |
| idratazione | 0 | 0 | 0 | 0 | 1 | 1 | 0 | 0 | 0 | 0 | .00055 | 1.60944 | 0 | 0 | 0 | 0 | .00089 |
| imbarazzo | 0 | 0 | 0 | 3 | 0 | 3 | 0 | 0 | 0 | .00168 | 0 | 1.60944 | 0 | 0 | 0 | .00270 | 0 |
| immagin* | 12 | 15 | 6 | 6 | 3 | 42 | .00496 | .01068 | .00604 | .00336 | .00166 | 0 | 0 | 0 | 0 | 0 | 0 |
| impaziente | 1 | 0 | 0 | 0 | 0 | 1 | .00041 | 0 | 0 | 0 | 0 | 1.60944 | .00066 | 0 | 0 | 0 | 0 |
| impazzisce | 0 | 1 | 0 | 0 | 0 | 1 | 0 | .00071 | 0 | 0 | 0 | 1.60944 | 0 | .00115 | 0 | 0 | 0 |
| impedi* | 1 | 1 | 0 | 0 | 1 | 3 | .00041 | .00071 | 0 | 0 | .00055 | .51083 | .00021 | .00036 | 0 | 0 | .00028 |
| imperf* | 0 | 0 | 0 | 0 | 2 | 2 | 0 | 0 | 0 | 0 | .00111 | 1.60944 | 0 | 0 | 0 | 0 | .00178 |
| impersonale | 1 | 0 | 0 | 0 | 0 | 1 | .00041 | 0 | 0 | 0 | 0 | 1.60944 | .00066 | 0 | 0 | 0 | 0 |
| impotenza | 2 | 0 | 0 | 0 | 0 | 2 | .00083 | 0 | 0 | 0 | 0 | 1.60944 | .00133 | 0 | 0 | 0 | 0 |
| impuls* | 0 | 0 | 1 | 0 | 1 | 2 | 0 | 0 | .00101 | 0 | .00055 | .91629 | 0 | 0 | .00092 | 0 | .00051 |
| inadeguat* | 1 | 0 | 0 | 0 | 2 | 3 | .00041 | 0 | 0 | 0 | .00111 | .91629 | .00038 | 0 | 0 | 0 | .00101 |
| inappagante | 1 | 0 | 0 | 0 | 0 | 1 | .00041 | 0 | 0 | 0 | 0 | 1.60944 | .00066 | 0 | 0 | 0 | 0 |
| inattività | 1 | 0 | 0 | 0 | 0 | 1 | .00041 | 0 | 0 | 0 | 0 | 1.60944 | .00066 | 0 | 0 | 0 | 0 |
| inclinazioni | 0 | 0 | 0 | 0 | 1 | 1 | 0 | 0 | 0 | 0 | .00055 | 1.60944 | 0 | 0 | 0 | 0 | .00089 |
| incontr* | 13 | 4 | 3 | 1 | 4 | 25 | .00537 | .00285 | .00302 | .00056 | .00221 | 0 | 0 | 0 | 0 | 0 | 0 |
| incuriosita | 0 | 0 | 0 | 0 | 1 | 1 | 0 | 0 | 0 | 0 | .00055 | 1.60944 | 0 | 0 | 0 | 0 | .00089 |
| indecisione | 1 | 0 | 0 | 0 | 0 | 1 | .00041 | 0 | 0 | 0 | 0 | 1.60944 | .00066 | 0 | 0 | 0 | 0 |
| indesiderato | 2 | 0 | 0 | 0 | 0 | 2 | .00083 | 0 | 0 | 0 | 0 | 1.60944 | .00133 | 0 | 0 | 0 | 0 |
| indifferente | 2 | 1 | 0 | 0 | 0 | 3 | .00083 | .00071 | 0 | 0 | 0 | .91629 | .00076 | .00065 | 0 | 0 | 0 |
| individual* | 12 | 0 | 3 | 14 | 1 | 30 | .00496 | 0 | .00302 | .00783 | .00055 | .22314 | .00111 | 0 | .00067 | .00175 | .00012 |
| indossabile | 0 | 0 | 0 | 1 | 0 | 1 | 0 | 0 | 0 | .00056 | 0 | 1.60944 | 0 | 0 | 0 | .00090 | 0 |
| indumenti | 0 | 0 | 0 | 1 | 0 | 1 | 0 | 0 | 0 | .00056 | 0 | 1.60944 | 0 | 0 | 0 | .00090 | 0 |
| inesaudito | 1 | 0 | 0 | 0 | 0 | 1 | .00041 | 0 | 0 | 0 | 0 | 1.60944 | .00066 | 0 | 0 | 0 | 0 |
| inesplorate | 1 | 0 | 0 | 0 | 0 | 1 | .00041 | 0 | 0 | 0 | 0 | 1.60944 | .00066 | 0 | 0 | 0 | 0 |
| inespresso | 1 | 1 | 0 | 0 | 0 | 2 | .00041 | .00071 | 0 | 0 | 0 | .91629 | .00038 | .00065 | 0 | 0 | 0 |
| inestetismi | 0 | 0 | 0 | 0 | 1 | 1 | 0 | 0 | 0 | 0 | .00055 | 1.60944 | 0 | 0 | 0 | 0 | .00089 |
| infastidiva | 0 | 0 | 0 | 0 | 1 | 1 | 0 | 0 | 0 | 0 | .00055 | 1.60944 | 0 | 0 | 0 | 0 | .00089 |
| ingabbiata | 0 | 0 | 0 | 0 | 1 | 1 | 0 | 0 | 0 | 0 | .00055 | 1.60944 | 0 | 0 | 0 | 0 | .00089 |
| ingegnati | 1 | 0 | 0 | 0 | 0 | 1 | .00041 | 0 | 0 | 0 | 0 | 1.60944 | .00066 | 0 | 0 | 0 | 0 |
| ingrassata | 0 | 1 | 0 | 0 | 0 | 1 | 0 | .00071 | 0 | 0 | 0 | 1.60944 | 0 | .00115 | 0 | 0 | 0 |
| ingrato | 1 | 0 | 0 | 0 | 0 | 1 | .00041 | 0 | 0 | 0 | 0 | 1.60944 | .00066 | 0 | 0 | 0 | 0 |
| ingrossata | 0 | 0 | 0 | 0 | 1 | 1 | 0 | 0 | 0 | 0 | .00055 | 1.60944 | 0 | 0 | 0 | 0 | .00089 |
| inibi* | 2 | 1 | 0 | 0 | 4 | 7 | .00083 | .00071 | 0 | 0 | .00221 | .51083 | .00042 | .00036 | 0 | 0 | .00113 |
| ininterrotto | 0 | 1 | 0 | 0 | 0 | 1 | 0 | .00071 | 0 | 0 | 0 | 1.60944 | 0 | .00115 | 0 | 0 | 0 |
| innamorat* | 0 | 1 | 0 | 0 | 3 | 4 | 0 | .00071 | 0 | 0 | .00166 | .91629 | 0 | .00065 | 0 | 0 | .00152 |
| innovativ* | 3 | 0 | 0 | 0 | 0 | 3 | .00124 | 0 | 0 | 0 | 0 | 1.60944 | .00199 | 0 | 0 | 0 | 0 |
| insapore | 1 | 0 | 0 | 0 | 0 | 1 | .00041 | 0 | 0 | 0 | 0 | 1.60944 | .00066 | 0 | 0 | 0 | 0 |
| insaziabile | 2 | 0 | 0 | 0 | 0 | 2 | .00083 | 0 | 0 | 0 | 0 | 1.60944 | .00133 | 0 | 0 | 0 | 0 |
| insegnamenti | 0 | 0 | 0 | 0 | 1 | 1 | 0 | 0 | 0 | 0 | .00055 | 1.60944 | 0 | 0 | 0 | 0 | .00089 |
| insicur* | 2 | 0 | 0 | 0 | 1 | 3 | .00083 | 0 | 0 | 0 | .00055 | .91629 | .00076 | 0 | 0 | 0 | .00051 |
| insoddisf* | 13 | 0 | 1 | 1 | 1 | 16 | .00537 | 0 | .00101 | .00056 | .00055 | .22314 | .00120 | 0 | .00022 | .00012 | .00012 |
| instagram | 0 | 1 | 0 | 0 | 0 | 1 | 0 | .00071 | 0 | 0 | 0 | 1.60944 | 0 | .00115 | 0 | 0 | 0 |
| instancabile | 1 | 0 | 0 | 0 | 0 | 1 | .00041 | 0 | 0 | 0 | 0 | 1.60944 | .00066 | 0 | 0 | 0 | 0 |
| intens* | 26 | 9 | 11 | 0 | 5 | 51 | .01074 | .00641 | .01108 | 0 | .00277 | .22314 | .00240 | .00143 | .00247 | 0 | .00062 |
| interazione | 1 | 0 | 0 | 1 | 1 | 3 | .00041 | 0 | 0 | .00056 | .00055 | .51083 | .00021 | 0 | 0 | .00029 | .00028 |
| interconnesse | 0 | 0 | 0 | 0 | 1 | 1 | 0 | 0 | 0 | 0 | .00055 | 1.60944 | 0 | 0 | 0 | 0 | .00089 |
| interior* | 0 | 0 | 0 | 0 | 2 | 2 | 0 | 0 | 0 | 0 | .00111 | 1.60944 | 0 | 0 | 0 | 0 | .00178 |
| internet | 0 | 3 | 1 | 9 | 1 | 14 | 0 | .00214 | .00101 | .00504 | .00055 | .22314 | 0 | .00048 | .00022 | .00112 | .00012 |
| interrotta | 4 | 0 | 1 | 1 | 0 | 6 | .00165 | 0 | .00101 | .00056 | 0 | .51083 | .00084 | 0 | .00051 | .00029 | 0 |
| intes* | 0 | 0 | 0 | 0 | 4 | 4 | 0 | 0 | 0 | 0 | .00221 | 1.60944 | 0 | 0 | 0 | 0 | .00356 |
| intim* | 23 | 4 | 4 | 3 | 14 | 48 | .00950 | .00285 | .00403 | .00168 | .00775 | 0 | 0 | 0 | 0 | 0 | 0 |
| intimorita | 1 | 0 | 0 | 0 | 0 | 1 | .00041 | 0 | 0 | 0 | 0 | 1.60944 | .00066 | 0 | 0 | 0 | 0 |
| intrig* | 4 | 1 | 0 | 1 | 0 | 6 | .00165 | .00071 | 0 | .00056 | 0 | .51083 | .00084 | .00036 | 0 | .00029 | 0 |
| introsp* | 1 | 0 | 0 | 0 | 1 | 2 | .00041 | 0 | 0 | 0 | .00055 | .91629 | .00038 | 0 | 0 | 0 | .00051 |
| invadente | 1 | 0 | 0 | 0 | 0 | 1 | .00041 | 0 | 0 | 0 | 0 | 1.60944 | .00066 | 0 | 0 | 0 | 0 |
| invecchiare | 0 | 0 | 0 | 0 | 1 | 1 | 0 | 0 | 0 | 0 | .00055 | 1.60944 | 0 | 0 | 0 | 0 | .00089 |
| invi* | 2 | 0 | 0 | 4 | 0 | 6 | .00083 | 0 | 0 | .00224 | 0 | .91629 | .00076 | 0 | 0 | .00205 | 0 |
| invogliato | 1 | 0 | 0 | 0 | 0 | 1 | .00041 | 0 | 0 | 0 | 0 | 1.60944 | .00066 | 0 | 0 | 0 | 0 |
| isol* | 4 | 0 | 2 | 0 | 1 | 7 | .00165 | 0 | .00201 | 0 | .00055 | .51083 | .00084 | 0 | .00103 | 0 | .00028 |
| isterico | 1 | 0 | 0 | 0 | 0 | 1 | .00041 | 0 | 0 | 0 | 0 | 1.60944 | .00066 | 0 | 0 | 0 | 0 |
| istint* | 1 | 1 | 0 | 0 | 0 | 2 | .00041 | .00071 | 0 | 0 | 0 | .91629 | .00038 | .00065 | 0 | 0 | 0 |
| latente | 0 | 0 | 1 | 0 | 0 | 1 | 0 | 0 | .00101 | 0 | 0 | 1.60944 | 0 | 0 | .00162 | 0 | 0 |
| lattice | 0 | 0 | 0 | 1 | 0 | 1 | 0 | 0 | 0 | .00056 | 0 | 1.60944 | 0 | 0 | 0 | .00090 | 0 |
| legame | 0 | 0 | 1 | 0 | 6 | 7 | 0 | 0 | .00101 | 0 | .00332 | .91629 | 0 | 0 | .00092 | 0 | .00304 |
| lesbo | 0 | 0 | 1 | 0 | 0 | 1 | 0 | 0 | .00101 | 0 | 0 | 1.60944 | 0 | 0 | .00162 | 0 | 0 |
| letargo | 1 | 0 | 0 | 0 | 0 | 1 | .00041 | 0 | 0 | 0 | 0 | 1.60944 | .00066 | 0 | 0 | 0 | 0 |
| lettura | 0 | 0 | 0 | 1 | 0 | 1 | 0 | 0 | 0 | .00056 | 0 | 1.60944 | 0 | 0 | 0 | .00090 | 0 |
| liber* | 18 | 10 | 9 | 2 | 20 | 59 | .00743 | .00712 | .00906 | .00112 | .01107 | 0 | 0 | 0 | 0 | 0 | 0 |
| libid* | 7 | 6 | 2 | 0 | 3 | 18 | .00289 | .00427 | .00201 | 0 | .00166 | .22314 | .00065 | .00095 | .00045 | 0 | .00037 |
| libri | 0 | 0 | 0 | 1 | 0 | 1 | 0 | 0 | 0 | .00056 | 0 | 1.60944 | 0 | 0 | 0 | .00090 | 0 |
| litig* | 2 | 0 | 0 | 0 | 0 | 2 | .00083 | 0 | 0 | 0 | 0 | 1.60944 | .00133 | 0 | 0 | 0 | 0 |
| live | 0 | 0 | 0 | 3 | 0 | 3 | 0 | 0 | 0 | .00168 | 0 | 1.60944 | 0 | 0 | 0 | .00270 | 0 |
| lontan* | 15 | 3 | 3 | 3 | 4 | 28 | .00620 | .00214 | .00302 | .00168 | .00221 | 0 | 0 | 0 | 0 | 0 | 0 |
| lubrificante | 0 | 0 | 0 | 2 | 0 | 2 | 0 | 0 | 0 | .00112 | 0 | 1.60944 | 0 | 0 | 0 | .00180 | 0 |
| lupoporno | 0 | 0 | 0 | 1 | 0 | 1 | 0 | 0 | 0 | .00056 | 0 | 1.60944 | 0 | 0 | 0 | .00090 | 0 |
| madre | 2 | 0 | 0 | 0 | 0 | 2 | .00083 | 0 | 0 | 0 | 0 | 1.60944 | .00133 | 0 | 0 | 0 | 0 |
| malessere | 1 | 0 | 0 | 0 | 0 | 1 | .00041 | 0 | 0 | 0 | 0 | 1.60944 | .00066 | 0 | 0 | 0 | 0 |
| malincon* | 5 | 0 | 1 | 0 | 0 | 6 | .00207 | 0 | .00101 | 0 | 0 | .91629 | .00189 | 0 | .00092 | 0 | 0 |
| malizioso | 1 | 0 | 0 | 0 | 0 | 1 | .00041 | 0 | 0 | 0 | 0 | 1.60944 | .00066 | 0 | 0 | 0 | 0 |
| mamma | 1 | 0 | 0 | 0 | 0 | 1 | .00041 | 0 | 0 | 0 | 0 | 1.60944 | .00066 | 0 | 0 | 0 | 0 |
| manc* | 47 | 10 | 10 | 3 | 28 | 98 | .01941 | .00712 | .01007 | .00168 | .01550 | 0 | 0 | 0 | 0 | 0 | 0 |
| mand* | 0 | 0 | 0 | 4 | 0 | 4 | 0 | 0 | 0 | .00224 | 0 | 1.60944 | 0 | 0 | 0 | .00360 | 0 |
| manga | 0 | 0 | 0 | 1 | 0 | 1 | 0 | 0 | 0 | .00056 | 0 | 1.60944 | 0 | 0 | 0 | .00090 | 0 |
| marijuana | 0 | 0 | 1 | 0 | 1 | 2 | 0 | 0 | .00101 | 0 | .00055 | .91629 | 0 | 0 | .00092 | 0 | .00051 |
| marito | 1 | 2 | 2 | 0 | 1 | 6 | .00041 | .00142 | .00201 | 0 | .00055 | .22314 | .00009 | .00032 | .00045 | 0 | .00012 |
| masch* | 0 | 0 | 1 | 1 | 1 | 3 | 0 | 0 | .00101 | .00056 | .00055 | .51083 | 0 | 0 | .00051 | .00029 | .00028 |
| masturb* | 56 | 6 | 165 | 20 | 39 | 286 | .02313 | .00427 | .16616 | .01119 | .02158 | 0 | 0 | 0 | 0 | 0 | 0 |
| menopausa | 0 | 0 | 1 | 0 | 0 | 1 | 0 | 0 | .00101 | 0 | 0 | 1.60944 | 0 | 0 | .00162 | 0 | 0 |
| messagg* | 3 | 2 | 3 | 18 | 1 | 27 | .00124 | .00142 | .00302 | .01007 | .00055 | 0 | 0 | 0 | 0 | 0 | 0 |
| mestrua* | 1 | 1 | 1 | 0 | 0 | 3 | .00041 | .00071 | .00101 | 0 | 0 | .51083 | .00021 | .00036 | .00051 | 0 | 0 |
| moglie | 0 | 1 | 0 | 0 | 0 | 1 | 0 | .00071 | 0 | 0 | 0 | 1.60944 | 0 | .00115 | 0 | 0 | 0 |
| monoton* | 9 | 0 | 2 | 1 | 1 | 13 | .00372 | 0 | .00201 | .00056 | .00055 | .22314 | .00083 | 0 | .00045 | .00012 | .00012 |
| morale | 1 | 0 | 0 | 0 | 0 | 1 | .00041 | 0 | 0 | 0 | 0 | 1.60944 | .00066 | 0 | 0 | 0 | 0 |
| morbida | 1 | 0 | 0 | 0 | 0 | 1 | .00041 | 0 | 0 | 0 | 0 | 1.60944 | .00066 | 0 | 0 | 0 | 0 |
| motiv* | 1 | 0 | 1 | 0 | 1 | 3 | .00041 | 0 | .00101 | 0 | .00055 | .51083 | .00021 | 0 | .00051 | 0 | .00028 |
| movimenti | 0 | 1 | 0 | 0 | 1 | 2 | 0 | .00071 | 0 | 0 | .00055 | .91629 | 0 | .00065 | 0 | 0 | .00051 |
| multimedial* | 1 | 0 | 0 | 1 | 0 | 2 | .00041 | 0 | 0 | .00056 | 0 | .91629 | .00038 | 0 | 0 | .00051 | 0 |
| nasco* | 3 | 2 | 0 | 1 | 0 | 6 | .00124 | .00142 | 0 | .00056 | 0 | .51083 | .00063 | .00073 | 0 | .00029 | 0 |
| navig* | 0 | 0 | 0 | 2 | 0 | 2 | 0 | 0 | 0 | .00112 | 0 | 1.60944 | 0 | 0 | 0 | .00180 | 0 |
| nervos* | 6 | 2 | 0 | 0 | 0 | 8 | .00248 | .00142 | 0 | 0 | 0 | .91629 | .00227 | .00131 | 0 | 0 | 0 |
| ninfomane | 1 | 0 | 0 | 0 | 0 | 1 | .00041 | 0 | 0 | 0 | 0 | 1.60944 | .00066 | 0 | 0 | 0 | 0 |
| noi* | 32 | 3 | 9 | 1 | 7 | 52 | .01322 | .00214 | .00906 | .00056 | .00387 | 0 | 0 | 0 | 0 | 0 | 0 |
| nostalgi* | 1 | 0 | 0 | 1 | 0 | 2 | .00041 | 0 | 0 | .00056 | 0 | .91629 | .00038 | 0 | 0 | .00051 | 0 |
| notturn* | 1 | 1 | 0 | 0 | 0 | 2 | .00041 | .00071 | 0 | 0 | 0 | .91629 | .00038 | .00065 | 0 | 0 | 0 |
| nud* | 0 | 0 | 1 | 2 | 2 | 5 | 0 | 0 | .00101 | .00112 | .00111 | .51083 | 0 | 0 | .00051 | .00057 | .00057 |
| nudes | 0 | 0 | 0 | 1 | 0 | 1 | 0 | 0 | 0 | .00056 | 0 | 1.60944 | 0 | 0 | 0 | .00090 | 0 |
| occasional* | 4 | 1 | 0 | 0 | 3 | 8 | .00165 | .00071 | 0 | 0 | .00166 | .51083 | .00084 | .00036 | 0 | 0 | .00085 |
| odio | 0 | 0 | 0 | 0 | 1 | 1 | 0 | 0 | 0 | 0 | .00055 | 1.60944 | 0 | 0 | 0 | 0 | .00089 |
| offendere | 1 | 0 | 0 | 0 | 0 | 1 | .00041 | 0 | 0 | 0 | 0 | 1.60944 | .00066 | 0 | 0 | 0 | 0 |
| ombelico | 0 | 0 | 0 | 0 | 1 | 1 | 0 | 0 | 0 | 0 | .00055 | 1.60944 | 0 | 0 | 0 | 0 | .00089 |
| omosessuali | 1 | 1 | 0 | 0 | 1 | 3 | .00041 | .00071 | 0 | 0 | .00055 | .51083 | .00021 | .00036 | 0 | 0 | .00028 |
| onanismo | 1 | 0 | 0 | 0 | 1 | 2 | .00041 | 0 | 0 | 0 | .00055 | .91629 | .00038 | 0 | 0 | 0 | .00051 |
| onirica | 1 | 0 | 0 | 0 | 1 | 2 | .00041 | 0 | 0 | 0 | .00055 | .91629 | .00038 | 0 | 0 | 0 | .00051 |
| online | 0 | 0 | 3 | 52 | 4 | 59 | 0 | 0 | .00302 | .02910 | .00221 | .51083 | 0 | 0 | .00154 | .01486 | .00113 |
| orale | 1 | 0 | 0 | 1 | 0 | 2 | .00041 | 0 | 0 | .00056 | 0 | .91629 | .00038 | 0 | 0 | .00051 | 0 |
| organi | 0 | 0 | 0 | 0 | 1 | 1 | 0 | 0 | 0 | 0 | .00055 | 1.60944 | 0 | 0 | 0 | 0 | .00089 |
| orgasm* | 5 | 2 | 6 | 1 | 7 | 21 | .00207 | .00142 | .00604 | .00056 | .00387 | 0 | 0 | 0 | 0 | 0 | 0 |
| orge | 0 | 1 | 0 | 0 | 0 | 1 | 0 | .00071 | 0 | 0 | 0 | 1.60944 | 0 | .00115 | 0 | 0 | 0 |
| orientamento | 0 | 0 | 0 | 0 | 3 | 3 | 0 | 0 | 0 | 0 | .00166 | 1.60944 | 0 | 0 | 0 | 0 | .00267 |
| ormon* | 3 | 3 | 0 | 0 | 1 | 7 | .00124 | .00214 | 0 | 0 | .00055 | .51083 | .00063 | .00109 | 0 | 0 | .00028 |
| osè | 0 | 0 | 0 | 1 | 0 | 1 | 0 | 0 | 0 | .00056 | 0 | 1.60944 | 0 | 0 | 0 | .00090 | 0 |
| pac* | 1 | 0 | 0 | 0 | 1 | 2 | .00041 | 0 | 0 | 0 | .00055 | .91629 | .00038 | 0 | 0 | 0 | .00051 |
| palliativi | 1 | 0 | 0 | 0 | 0 | 1 | .00041 | 0 | 0 | 0 | 0 | 1.60944 | .00066 | 0 | 0 | 0 | 0 |
| palline | 0 | 0 | 0 | 1 | 0 | 1 | 0 | 0 | 0 | .00056 | 0 | 1.60944 | 0 | 0 | 0 | .00090 | 0 |
| paranoie | 0 | 0 | 0 | 0 | 1 | 1 | 0 | 0 | 0 | 0 | .00055 | 1.60944 | 0 | 0 | 0 | 0 | .00089 |
| parente | 1 | 0 | 0 | 0 | 0 | 1 | .00041 | 0 | 0 | 0 | 0 | 1.60944 | .00066 | 0 | 0 | 0 | 0 |
| parl* | 5 | 4 | 1 | 3 | 5 | 18 | .00207 | .00285 | .00101 | .00168 | .00277 | 0 | 0 | 0 | 0 | 0 | 0 |
| parole | 3 | 1 | 0 | 0 | 1 | 5 | .00124 | .00071 | 0 | 0 | .00055 | .51083 | .00063 | .00036 | 0 | 0 | .00028 |
| partecip* | 0 | 3 | 0 | 0 | 0 | 3 | 0 | .00214 | 0 | 0 | 0 | 1.60944 | 0 | .00344 | 0 | 0 | 0 |
| partner | 122 | 51 | 51 | 96 | 88 | 408 | .05039 | .03632 | .05136 | .05372 | .04870 | 0 | 0 | 0 | 0 | 0 | 0 |
| passion* | 17 | 2 | 1 | 0 | 4 | 24 | .00702 | .00142 | .00101 | 0 | .00221 | .22314 | .00157 | .00032 | .00022 | 0 | .00049 |
| passiv* | 0 | 2 | 0 | 0 | 0 | 2 | 0 | .00142 | 0 | 0 | 0 | 1.60944 | 0 | .00229 | 0 | 0 | 0 |
| paur* | 9 | 3 | 1 | 0 | 5 | 18 | .00372 | .00214 | .00101 | 0 | .00277 | .22314 | .00083 | .00048 | .00022 | 0 | .00062 |
| pazienza | 0 | 0 | 0 | 0 | 2 | 2 | 0 | 0 | 0 | 0 | .00111 | 1.60944 | 0 | 0 | 0 | 0 | .00178 |
| pc | 0 | 0 | 0 | 1 | 0 | 1 | 0 | 0 | 0 | .00056 | 0 | 1.60944 | 0 | 0 | 0 | .00090 | 0 |
| pelle | 3 | 0 | 0 | 2 | 0 | 5 | .00124 | 0 | 0 | .00112 | 0 | .91629 | .00114 | 0 | 0 | .00103 | 0 |
| pene | 0 | 1 | 0 | 0 | 1 | 2 | 0 | .00071 | 0 | 0 | .00055 | .91629 | 0 | .00065 | 0 | 0 | .00051 |
| penetrazione | 1 | 0 | 0 | 0 | 1 | 2 | .00041 | 0 | 0 | 0 | .00055 | .91629 | .00038 | 0 | 0 | 0 | .00051 |
| percezione | 2 | 0 | 0 | 0 | 1 | 3 | .00083 | 0 | 0 | 0 | .00055 | .91629 | .00076 | 0 | 0 | 0 | .00051 |
| performance | 2 | 0 | 0 | 0 | 0 | 2 | .00083 | 0 | 0 | 0 | 0 | 1.60944 | .00133 | 0 | 0 | 0 | 0 |
| pervers* | 2 | 3 | 1 | 0 | 1 | 7 | .00083 | .00214 | .00101 | 0 | .00055 | .22314 | .00018 | .00048 | .00022 | 0 | .00012 |
| petting | 1 | 0 | 0 | 0 | 0 | 1 | .00041 | 0 | 0 | 0 | 0 | 1.60944 | .00066 | 0 | 0 | 0 | 0 |
| piac* | 37 | 5 | 20 | 13 | 75 | 150 | .01528 | .00356 | .02014 | .00727 | .04151 | 0 | 0 | 0 | 0 | 0 | 0 |
| piangere | 0 | 1 | 0 | 0 | 0 | 1 | 0 | .00071 | 0 | 0 | 0 | 1.60944 | 0 | .00115 | 0 | 0 | 0 |
| piattaform* | 0 | 0 | 2 | 1 | 0 | 3 | 0 | 0 | .00201 | .00056 | 0 | .91629 | 0 | 0 | .00185 | .00051 | 0 |
| piccanti | 0 | 1 | 0 | 1 | 1 | 3 | 0 | .00071 | 0 | .00056 | .00055 | .51083 | 0 | .00036 | 0 | .00029 | .00028 |
| pigrizia | 0 | 0 | 0 | 1 | 0 | 1 | 0 | 0 | 0 | .00056 | 0 | 1.60944 | 0 | 0 | 0 | .00090 | 0 |
| pillol* | 1 | 1 | 1 | 0 | 0 | 3 | .00041 | .00071 | .00101 | 0 | 0 | .51083 | .00021 | .00036 | .00051 | 0 | 0 |
| placebo | 0 | 0 | 1 | 0 | 0 | 1 | 0 | 0 | .00101 | 0 | 0 | 1.60944 | 0 | 0 | .00162 | 0 | 0 |
| pornhub | 1 | 0 | 1 | 5 | 0 | 7 | .00041 | 0 | .00101 | .00280 | 0 | .51083 | .00021 | 0 | .00051 | .00143 | 0 |
| porno* | 9 | 5 | 11 | 59 | 9 | 93 | .00372 | .00356 | .01108 | .03302 | .00498 | 0 | 0 | 0 | 0 | 0 | 0 |
| posizion* | 2 | 1 | 0 | 0 | 3 | 6 | .00083 | .00071 | 0 | 0 | .00166 | .51083 | .00042 | .00036 | 0 | 0 | .00085 |
| precauzioni | 1 | 1 | 0 | 0 | 1 | 3 | .00041 | .00071 | 0 | 0 | .00055 | .51083 | .00021 | .00036 | 0 | 0 | .00028 |
| preliminari | 1 | 0 | 0 | 0 | 6 | 7 | .00041 | 0 | 0 | 0 | .00332 | .91629 | .00038 | 0 | 0 | 0 | .00304 |
| premura | 0 | 0 | 1 | 0 | 0 | 1 | 0 | 0 | .00101 | 0 | 0 | 1.60944 | 0 | 0 | .00162 | 0 | 0 |
| preoccup* | 10 | 1 | 2 | 0 | 2 | 15 | .00413 | .00071 | .00201 | 0 | .00111 | .22314 | .00092 | .00016 | .00045 | 0 | .00025 |
| privacy | 5 | 0 | 8 | 1 | 0 | 14 | .00207 | 0 | .00806 | .00056 | 0 | .51083 | .00105 | 0 | .00412 | .00029 | 0 |
| promiscu* | 1 | 0 | 0 | 0 | 1 | 2 | .00041 | 0 | 0 | 0 | .00055 | .91629 | .00038 | 0 | 0 | 0 | .00051 |
| provoc* | 4 | 0 | 0 | 3 | 2 | 9 | .00165 | 0 | 0 | .00168 | .00111 | .51083 | .00084 | 0 | 0 | .00086 | .00057 |
| pudore | 0 | 0 | 0 | 0 | 1 | 1 | 0 | 0 | 0 | 0 | .00055 | 1.60944 | 0 | 0 | 0 | 0 | .00089 |
| pulsion* | 1 | 3 | 1 | 0 | 4 | 9 | .00041 | .00214 | .00101 | 0 | .00221 | .22314 | .00009 | .00048 | .00022 | 0 | .00049 |
| quotidian* | 6 | 4 | 6 | 3 | 8 | 27 | .00248 | .00285 | .00604 | .00168 | .00443 | 0 | 0 | 0 | 0 | 0 | 0 |
| rabbia | 2 | 1 | 0 | 0 | 1 | 4 | .00083 | .00071 | 0 | 0 | .00055 | .51083 | .00042 | .00036 | 0 | 0 | .00028 |
| racconti | 0 | 0 | 1 | 5 | 0 | 6 | 0 | 0 | .00101 | .00280 | 0 | .91629 | 0 | 0 | .00092 | .00256 | 0 |
| ragazz* | 28 | 9 | 11 | 12 | 10 | 70 | .01157 | .00641 | .01108 | .00672 | .00553 | 0 | 0 | 0 | 0 | 0 | 0 |
| rapport* | 61 | 13 | 14 | 11 | 62 | 161 | .02520 | .00926 | .01410 | .00616 | .03431 | 0 | 0 | 0 | 0 | 0 | 0 |
| rassegn* | 1 | 1 | 0 | 0 | 0 | 2 | .00041 | .00071 | 0 | 0 | 0 | .91629 | .00038 | .00065 | 0 | 0 | 0 |
| reinvent* | 2 | 0 | 0 | 0 | 0 | 2 | .00083 | 0 | 0 | 0 | 0 | 1.60944 | .00133 | 0 | 0 | 0 | 0 |
| relax | 0 | 0 | 0 | 0 | 1 | 1 | 0 | 0 | 0 | 0 | .00055 | 1.60944 | 0 | 0 | 0 | 0 | .00089 |
| relazion* | 15 | 4 | 0 | 3 | 10 | 32 | .00620 | .00285 | 0 | .00168 | .00553 | .22314 | .00138 | .00064 | 0 | .00037 | .00123 |
| repress* | 4 | 0 | 0 | 0 | 0 | 4 | .00165 | 0 | 0 | 0 | 0 | 1.60944 | .00266 | 0 | 0 | 0 | 0 |
| repulsione | 1 | 0 | 0 | 0 | 0 | 1 | .00041 | 0 | 0 | 0 | 0 | 1.60944 | .00066 | 0 | 0 | 0 | 0 |
| riavvicinarmi | 0 | 0 | 0 | 0 | 1 | 1 | 0 | 0 | 0 | 0 | .00055 | 1.60944 | 0 | 0 | 0 | 0 | .00089 |
| ricatto | 0 | 0 | 0 | 0 | 1 | 1 | 0 | 0 | 0 | 0 | .00055 | 1.60944 | 0 | 0 | 0 | 0 | .00089 |
| ricerc* | 11 | 5 | 5 | 4 | 4 | 29 | .00454 | .00356 | .00504 | .00224 | .00221 | 0 | 0 | 0 | 0 | 0 | 0 |
| riconciliante | 1 | 0 | 0 | 0 | 0 | 1 | .00041 | 0 | 0 | 0 | 0 | 1.60944 | .00066 | 0 | 0 | 0 | 0 |
| ricongiun* | 3 | 1 | 0 | 0 | 0 | 4 | .00124 | .00071 | 0 | 0 | 0 | .91629 | .00114 | .00065 | 0 | 0 | 0 |
| ricontattare | 0 | 0 | 0 | 1 | 0 | 1 | 0 | 0 | 0 | .00056 | 0 | 1.60944 | 0 | 0 | 0 | .00090 | 0 |
| ricreative | 0 | 1 | 0 | 0 | 0 | 1 | 0 | .00071 | 0 | 0 | 0 | 1.60944 | 0 | .00115 | 0 | 0 | 0 |
| rigenerante | 1 | 0 | 0 | 0 | 0 | 1 | .00041 | 0 | 0 | 0 | 0 | 1.60944 | .00066 | 0 | 0 | 0 | 0 |
| rilasciare | 0 | 0 | 1 | 0 | 0 | 1 | 0 | 0 | .00101 | 0 | 0 | 1.60944 | 0 | 0 | .00162 | 0 | 0 |
| rilass* | 18 | 1 | 10 | 0 | 3 | 32 | .00743 | .00071 | .01007 | 0 | .00166 | .22314 | .00166 | .00016 | .00225 | 0 | .00037 |
| ring | 0 | 0 | 0 | 1 | 0 | 1 | 0 | 0 | 0 | .00056 | 0 | 1.60944 | 0 | 0 | 0 | .00090 | 0 |
| riposo | 0 | 1 | 0 | 0 | 1 | 2 | 0 | .00071 | 0 | 0 | .00055 | .91629 | 0 | .00065 | 0 | 0 | .00051 |
| rischi* | 1 | 0 | 0 | 0 | 2 | 3 | .00041 | 0 | 0 | 0 | .00111 | .91629 | .00038 | 0 | 0 | 0 | .00101 |
| riscop* | 4 | 2 | 1 | 1 | 6 | 14 | .00165 | .00142 | .00101 | .00056 | .00332 | 0 | 0 | 0 | 0 | 0 | 0 |
| risollev* | 2 | 0 | 0 | 0 | 0 | 2 | .00083 | 0 | 0 | 0 | 0 | 1.60944 | .00133 | 0 | 0 | 0 | 0 |
| risvegl* | 3 | 3 | 1 | 0 | 0 | 7 | .00124 | .00214 | .00101 | 0 | 0 | .51083 | .00063 | .00109 | .00051 | 0 | 0 |
| riuniti | 0 | 1 | 0 | 0 | 0 | 1 | 0 | .00071 | 0 | 0 | 0 | 1.60944 | 0 | .00115 | 0 | 0 | 0 |
| rived* | 4 | 4 | 0 | 0 | 0 | 8 | .00165 | .00285 | 0 | 0 | 0 | .91629 | .00151 | .00261 | 0 | 0 | 0 |
| role | 0 | 0 | 0 | 1 | 0 | 1 | 0 | 0 | 0 | .00056 | 0 | 1.60944 | 0 | 0 | 0 | .00090 | 0 |
| romantic* | 2 | 1 | 0 | 1 | 1 | 5 | .00083 | .00071 | 0 | .00056 | .00055 | .22314 | .00018 | .00016 | 0 | .00012 | .00012 |
| romanzi | 0 | 0 | 0 | 1 | 0 | 1 | 0 | 0 | 0 | .00056 | 0 | 1.60944 | 0 | 0 | 0 | .00090 | 0 |
| rottura | 1 | 0 | 0 | 0 | 0 | 1 | .00041 | 0 | 0 | 0 | 0 | 1.60944 | .00066 | 0 | 0 | 0 | 0 |
| routin* | 2 | 0 | 2 | 0 | 6 | 10 | .00083 | 0 | .00201 | 0 | .00332 | .51083 | .00042 | 0 | .00103 | 0 | .00170 |
| salut* | 1 | 1 | 1 | 0 | 4 | 7 | .00041 | .00071 | .00101 | 0 | .00221 | .22314 | .00009 | .00016 | .00022 | 0 | .00049 |
| san* | 2 | 0 | 1 | 0 | 2 | 5 | .00083 | 0 | .00101 | 0 | .00111 | .51083 | .00042 | 0 | .00051 | 0 | .00057 |
| sanguigno | 0 | 0 | 0 | 0 | 1 | 1 | 0 | 0 | 0 | 0 | .00055 | 1.60944 | 0 | 0 | 0 | 0 | .00089 |
| saziati | 1 | 0 | 0 | 0 | 0 | 1 | .00041 | 0 | 0 | 0 | 0 | 1.60944 | .00066 | 0 | 0 | 0 | 0 |
| sbloccarmi | 0 | 0 | 0 | 0 | 2 | 2 | 0 | 0 | 0 | 0 | .00111 | 1.60944 | 0 | 0 | 0 | 0 | .00178 |
| sbronz* | 0 | 0 | 2 | 0 | 0 | 2 | 0 | 0 | .00201 | 0 | 0 | 1.60944 | 0 | 0 | .00324 | 0 | 0 |
| scaric* | 1 | 1 | 0 | 0 | 4 | 6 | .00041 | .00071 | 0 | 0 | .00221 | .51083 | .00021 | .00036 | 0 | 0 | .00113 |
| sciogliermi | 0 | 0 | 0 | 0 | 1 | 1 | 0 | 0 | 0 | 0 | .00055 | 1.60944 | 0 | 0 | 0 | 0 | .00089 |
| sciolta | 0 | 0 | 0 | 0 | 1 | 1 | 0 | 0 | 0 | 0 | .00055 | 1.60944 | 0 | 0 | 0 | 0 | .00089 |
| scomod* | 1 | 0 | 1 | 0 | 1 | 3 | .00041 | 0 | .00101 | 0 | .00055 | .51083 | .00021 | 0 | .00051 | 0 | .00028 |
| scompensi | 0 | 1 | 0 | 0 | 0 | 1 | 0 | .00071 | 0 | 0 | 0 | 1.60944 | 0 | .00115 | 0 | 0 | 0 |
| sconfort* | 3 | 2 | 0 | 0 | 0 | 5 | .00124 | .00142 | 0 | 0 | 0 | .91629 | .00114 | .00131 | 0 | 0 | 0 |
| sconosciut* | 0 | 2 | 0 | 2 | 1 | 5 | 0 | .00142 | 0 | .00112 | .00055 | .51083 | 0 | .00073 | 0 | .00057 | .00028 |
| sconvolg* | 1 | 0 | 0 | 0 | 1 | 2 | .00041 | 0 | 0 | 0 | .00055 | .91629 | .00038 | 0 | 0 | 0 | .00051 |
| scopare | 0 | 0 | 0 | 0 | 1 | 1 | 0 | 0 | 0 | 0 | .00055 | 1.60944 | 0 | 0 | 0 | 0 | .00089 |
| scoraggiante | 1 | 0 | 0 | 0 | 0 | 1 | .00041 | 0 | 0 | 0 | 0 | 1.60944 | .00066 | 0 | 0 | 0 | 0 |
| scritt* | 3 | 0 | 0 | 1 | 0 | 4 | .00124 | 0 | 0 | .00056 | 0 | .91629 | .00114 | 0 | 0 | .00051 | 0 |
| scriv* | 0 | 0 | 0 | 2 | 0 | 2 | 0 | 0 | 0 | .00112 | 0 | 1.60944 | 0 | 0 | 0 | .00180 | 0 |
| secrezione | 1 | 0 | 0 | 0 | 0 | 1 | .00041 | 0 | 0 | 0 | 0 | 1.60944 | .00066 | 0 | 0 | 0 | 0 |
| sedurre | 1 | 0 | 0 | 0 | 0 | 1 | .00041 | 0 | 0 | 0 | 0 | 1.60944 | .00066 | 0 | 0 | 0 | 0 |
| segret* | 2 | 1 | 0 | 0 | 0 | 3 | .00083 | .00071 | 0 | 0 | 0 | .91629 | .00076 | .00065 | 0 | 0 | 0 |
| selvaggio | 0 | 1 | 0 | 0 | 0 | 1 | 0 | .00071 | 0 | 0 | 0 | 1.60944 | 0 | .00115 | 0 | 0 | 0 |
| sens* | 25 | 5 | 5 | 4 | 66 | 105 | .01033 | .00356 | .00504 | .00224 | .03652 | 0 | 0 | 0 | 0 | 0 | 0 |
| sensibil* | 1 | 0 | 2 | 0 | 1 | 4 | .00041 | 0 | .00201 | 0 | .00055 | .51083 | .00021 | 0 | .00103 | 0 | .00028 |
| sensual* | 0 | 0 | 0 | 1 | 3 | 4 | 0 | 0 | 0 | .00056 | .00166 | .91629 | 0 | 0 | 0 | .00051 | .00152 |
| sentiment* | 5 | 1 | 0 | 0 | 1 | 7 | .00207 | .00071 | 0 | 0 | .00055 | .51083 | .00105 | .00036 | 0 | 0 | .00028 |
| separ* | 10 | 2 | 1 | 1 | 4 | 18 | .00413 | .00142 | .00101 | .00056 | .00221 | 0 | 0 | 0 | 0 | 0 | 0 |
| seren* | 8 | 0 | 3 | 1 | 6 | 18 | .00330 | 0 | .00302 | .00056 | .00332 | .22314 | .00074 | 0 | .00067 | .00012 | .00074 |
| sess* | 221 | 109 | 33 | 171 | 256 | 790 | .09128 | .07764 | .03323 | .09569 | .14167 | 0 | 0 | 0 | 0 | 0 | 0 |
| sex | 2 | 4 | 10 | 159 | 11 | 186 | .00083 | .00285 | .01007 | .08898 | .00609 | 0 | 0 | 0 | 0 | 0 | 0 |
| sexting | 6 | 2 | 4 | 85 | 10 | 107 | .00248 | .00142 | .00403 | .04757 | .00553 | 0 | 0 | 0 | 0 | 0 | 0 |
| sextortion | 0 | 0 | 0 | 1 | 0 | 1 | 0 | 0 | 0 | .00056 | 0 | 1.60944 | 0 | 0 | 0 | .00090 | 0 |
| sexy | 0 | 1 | 0 | 1 | 1 | 3 | 0 | .00071 | 0 | .00056 | .00055 | .51083 | 0 | .00036 | 0 | .00029 | .00028 |
| sfog* | 3 | 2 | 4 | 0 | 6 | 15 | .00124 | .00142 | .00403 | 0 | .00332 | .22314 | .00028 | .00032 | .00090 | 0 | .00074 |
| sforzo | 1 | 0 | 0 | 0 | 0 | 1 | .00041 | 0 | 0 | 0 | 0 | 1.60944 | .00066 | 0 | 0 | 0 | 0 |
| sfrenata | 1 | 0 | 0 | 0 | 0 | 1 | .00041 | 0 | 0 | 0 | 0 | 1.60944 | .00066 | 0 | 0 | 0 | 0 |
| sgradevoli | 0 | 0 | 0 | 0 | 1 | 1 | 0 | 0 | 0 | 0 | .00055 | 1.60944 | 0 | 0 | 0 | 0 | .00089 |
| single | 7 | 0 | 3 | 1 | 2 | 13 | .00289 | 0 | .00302 | .00056 | .00111 | .22314 | .00065 | 0 | .00067 | .00012 | .00025 |
| sintonia | 2 | 1 | 0 | 0 | 1 | 4 | .00083 | .00071 | 0 | 0 | .00055 | .51083 | .00042 | .00036 | 0 | 0 | .00028 |
| sit* | 3 | 2 | 1 | 123 | 6 | 135 | .00124 | .00142 | .00101 | .06883 | .00332 | 0 | 0 | 0 | 0 | 0 | 0 |
| skype | 2 | 0 | 0 | 1 | 0 | 3 | .00083 | 0 | 0 | .00056 | 0 | .91629 | .00076 | 0 | 0 | .00051 | 0 |
| smartphone | 1 | 0 | 0 | 1 | 0 | 2 | .00041 | 0 | 0 | .00056 | 0 | .91629 | .00038 | 0 | 0 | .00051 | 0 |
| sms | 0 | 1 | 0 | 0 | 0 | 1 | 0 | .00071 | 0 | 0 | 0 | 1.60944 | 0 | .00115 | 0 | 0 | 0 |
| snervante | 1 | 0 | 0 | 0 | 0 | 1 | .00041 | 0 | 0 | 0 | 0 | 1.60944 | .00066 | 0 | 0 | 0 | 0 |
| social* | 6 | 1 | 0 | 0 | 2 | 9 | .00248 | .00071 | 0 | 0 | .00111 | .51083 | .00127 | .00036 | 0 | 0 | .00057 |
| società | 0 | 0 | 0 | 1 | 0 | 1 | 0 | 0 | 0 | .00056 | 0 | 1.60944 | 0 | 0 | 0 | .00090 | 0 |
| soddisf* | 33 | 5 | 16 | 4 | 21 | 79 | .01363 | .00356 | .01611 | .00224 | .01162 | 0 | 0 | 0 | 0 | 0 | 0 |
| soff* | 6 | 0 | 0 | 0 | 0 | 6 | .00248 | 0 | 0 | 0 | 0 | 1.60944 | .00399 | 0 | 0 | 0 | 0 |
| sogn* | 9 | 13 | 0 | 0 | 4 | 26 | .00372 | .00926 | 0 | 0 | .00221 | .51083 | .00190 | .00473 | 0 | 0 | .00113 |
| solitari* | 34 | 1 | 2 | 1 | 1 | 39 | .01404 | .00071 | .00201 | .00056 | .00055 | 0 | 0 | 0 | 0 | 0 | 0 |
| solitudine | 9 | 0 | 5 | 0 | 3 | 17 | .00372 | 0 | .00504 | 0 | .00166 | .51083 | .00190 | 0 | .00257 | 0 | .00085 |
| sollievo | 2 | 0 | 0 | 0 | 0 | 2 | .00083 | 0 | 0 | 0 | 0 | 1.60944 | .00133 | 0 | 0 | 0 | 0 |
| somatizzato | 0 | 1 | 0 | 0 | 0 | 1 | 0 | .00071 | 0 | 0 | 0 | 1.60944 | 0 | .00115 | 0 | 0 | 0 |
| sonnifero | 0 | 0 | 1 | 0 | 0 | 1 | 0 | 0 | .00101 | 0 | 0 | 1.60944 | 0 | 0 | .00162 | 0 | 0 |
| sopport* | 2 | 0 | 0 | 0 | 1 | 3 | .00083 | 0 | 0 | 0 | .00055 | .91629 | .00076 | 0 | 0 | 0 | .00051 |
| sorella | 0 | 0 | 2 | 0 | 0 | 2 | 0 | 0 | .00201 | 0 | 0 | 1.60944 | 0 | 0 | .00324 | 0 | 0 |
| sorpr* | 4 | 1 | 0 | 1 | 0 | 6 | .00165 | .00071 | 0 | .00056 | 0 | .51083 | .00084 | .00036 | 0 | .00029 | 0 |
| sostegno | 0 | 0 | 0 | 0 | 1 | 1 | 0 | 0 | 0 | 0 | .00055 | 1.60944 | 0 | 0 | 0 | 0 | .00089 |
| sottomessa | 0 | 1 | 0 | 0 | 0 | 1 | 0 | .00071 | 0 | 0 | 0 | 1.60944 | 0 | .00115 | 0 | 0 | 0 |
| sottomissione | 0 | 1 | 0 | 0 | 1 | 2 | 0 | .00071 | 0 | 0 | .00055 | .91629 | 0 | .00065 | 0 | 0 | .00051 |
| sottotono | 0 | 0 | 1 | 0 | 0 | 1 | 0 | 0 | .00101 | 0 | 0 | 1.60944 | 0 | 0 | .00162 | 0 | 0 |
| spavento | 0 | 1 | 0 | 0 | 0 | 1 | 0 | .00071 | 0 | 0 | 0 | 1.60944 | 0 | .00115 | 0 | 0 | 0 |
| spensierat* | 2 | 0 | 0 | 0 | 1 | 3 | .00083 | 0 | 0 | 0 | .00055 | .91629 | .00076 | 0 | 0 | 0 | .00051 |
| speranza | 1 | 0 | 0 | 0 | 0 | 1 | .00041 | 0 | 0 | 0 | 0 | 1.60944 | .00066 | 0 | 0 | 0 | 0 |
| speriment* | 25 | 11 | 10 | 5 | 16 | 67 | .01033 | .00783 | .01007 | .00280 | .00885 | 0 | 0 | 0 | 0 | 0 | 0 |
| spiacevol* | 2 | 0 | 0 | 0 | 0 | 2 | .00083 | 0 | 0 | 0 | 0 | 1.60944 | .00133 | 0 | 0 | 0 | 0 |
| spint* | 2 | 5 | 1 | 3 | 0 | 11 | .00083 | .00356 | .00101 | .00168 | 0 | .22314 | .00018 | .00079 | .00022 | .00037 | 0 |
| spontane* | 3 | 1 | 1 | 0 | 3 | 8 | .00124 | .00071 | .00101 | 0 | .00166 | .22314 | .00028 | .00016 | .00022 | 0 | .00037 |
| sporca | 0 | 0 | 0 | 0 | 2 | 2 | 0 | 0 | 0 | 0 | .00111 | 1.60944 | 0 | 0 | 0 | 0 | .00178 |
| squirtare | 0 | 0 | 0 | 0 | 1 | 1 | 0 | 0 | 0 | 0 | .00055 | 1.60944 | 0 | 0 | 0 | 0 | .00089 |
| stanc* | 8 | 1 | 1 | 0 | 1 | 11 | .00330 | .00071 | .00101 | 0 | .00055 | .22314 | .00074 | .00016 | .00022 | 0 | .00012 |
| stereotipi | 0 | 0 | 0 | 0 | 1 | 1 | 0 | 0 | 0 | 0 | .00055 | 1.60944 | 0 | 0 | 0 | 0 | .00089 |
| stimol* | 27 | 11 | 8 | 5 | 8 | 59 | .01115 | .00783 | .00806 | .00280 | .00443 | 0 | 0 | 0 | 0 | 0 | 0 |
| stravolto | 0 | 0 | 0 | 0 | 1 | 1 | 0 | 0 | 0 | 0 | .00055 | 1.60944 | 0 | 0 | 0 | 0 | .00089 |
| straziante | 1 | 0 | 0 | 0 | 0 | 1 | .00041 | 0 | 0 | 0 | 0 | 1.60944 | .00066 | 0 | 0 | 0 | 0 |
| streaming | 0 | 0 | 0 | 1 | 0 | 1 | 0 | 0 | 0 | .00056 | 0 | 1.60944 | 0 | 0 | 0 | .00090 | 0 |
| stress* | 13 | 3 | 6 | 0 | 7 | 29 | .00537 | .00214 | .00604 | 0 | .00387 | .22314 | .00120 | .00048 | .00135 | 0 | .00086 |
| strument* | 0 | 0 | 0 | 7 | 2 | 9 | 0 | 0 | 0 | .00392 | .00111 | .91629 | 0 | 0 | 0 | .00359 | .00101 |
| strusciavo | 0 | 0 | 0 | 1 | 0 | 1 | 0 | 0 | 0 | .00056 | 0 | 1.60944 | 0 | 0 | 0 | .00090 | 0 |
| stufandomi | 0 | 0 | 1 | 0 | 0 | 1 | 0 | 0 | .00101 | 0 | 0 | 1.60944 | 0 | 0 | .00162 | 0 | 0 |
| stuzzicati | 1 | 0 | 0 | 0 | 0 | 1 | .00041 | 0 | 0 | 0 | 0 | 1.60944 | .00066 | 0 | 0 | 0 | 0 |
| surrog* | 0 | 0 | 1 | 0 | 1 | 2 | 0 | 0 | .00101 | 0 | .00055 | .91629 | 0 | 0 | .00092 | 0 | .00051 |
| svago | 0 | 1 | 2 | 0 | 0 | 3 | 0 | .00071 | .00201 | 0 | 0 | .91629 | 0 | .00065 | .00185 | 0 | 0 |
| svogliat* | 4 | 0 | 0 | 0 | 0 | 4 | .00165 | 0 | 0 | 0 | 0 | 1.60944 | .00266 | 0 | 0 | 0 | 0 |
| svuot* | 0 | 0 | 1 | 0 | 2 | 3 | 0 | 0 | .00101 | 0 | .00111 | .91629 | 0 | 0 | .00092 | 0 | .00101 |
| taboo | 0 | 0 | 0 | 0 | 1 | 1 | 0 | 0 | 0 | 0 | .00055 | 1.60944 | 0 | 0 | 0 | 0 | .00089 |
| tabù | 0 | 0 | 0 | 0 | 1 | 1 | 0 | 0 | 0 | 0 | .00055 | 1.60944 | 0 | 0 | 0 | 0 | .00089 |
| tatto | 0 | 1 | 0 | 0 | 1 | 2 | 0 | .00071 | 0 | 0 | .00055 | .91629 | 0 | .00065 | 0 | 0 | .00051 |
| tecniche | 0 | 0 | 0 | 1 | 1 | 2 | 0 | 0 | 0 | .00056 | .00055 | .91629 | 0 | 0 | 0 | .00051 | .00051 |
| tecnolog* | 1 | 0 | 0 | 45 | 1 | 47 | .00041 | 0 | 0 | .02518 | .00055 | .51083 | .00021 | 0 | 0 | .01286 | .00028 |
| telefon* | 2 | 1 | 6 | 9 | 1 | 19 | .00083 | .00071 | .00604 | .00504 | .00055 | 0 | 0 | 0 | 0 | 0 | 0 |
| telematic* | 1 | 0 | 1 | 0 | 0 | 2 | .00041 | 0 | .00101 | 0 | 0 | .91629 | .00038 | 0 | .00092 | 0 | 0 |
| temere | 0 | 0 | 1 | 0 | 0 | 1 | 0 | 0 | .00101 | 0 | 0 | 1.60944 | 0 | 0 | .00162 | 0 | 0 |
| tenerezza | 0 | 1 | 0 | 0 | 6 | 7 | 0 | .00071 | 0 | 0 | .00332 | .91629 | 0 | .00065 | 0 | 0 | .00304 |
| tenero | 2 | 0 | 0 | 0 | 0 | 2 | .00083 | 0 | 0 | 0 | 0 | 1.60944 | .00133 | 0 | 0 | 0 | 0 |
| tension* | 2 | 1 | 0 | 0 | 2 | 5 | .00083 | .00071 | 0 | 0 | .00111 | .51083 | .00042 | .00036 | 0 | 0 | .00057 |
| tentazione | 0 | 0 | 0 | 1 | 0 | 1 | 0 | 0 | 0 | .00056 | 0 | 1.60944 | 0 | 0 | 0 | .00090 | 0 |
| tesa | 0 | 0 | 2 | 0 | 0 | 2 | 0 | 0 | .00201 | 0 | 0 | 1.60944 | 0 | 0 | .00324 | 0 | 0 |
| testa | 1 | 2 | 2 | 0 | 1 | 6 | .00041 | .00142 | .00201 | 0 | .00055 | .22314 | .00009 | .00032 | .00045 | 0 | .00012 |
| testi | 1 | 0 | 0 | 0 | 0 | 1 | .00041 | 0 | 0 | 0 | 0 | 1.60944 | .00066 | 0 | 0 | 0 | 0 |
| threesome | 0 | 1 | 0 | 0 | 0 | 1 | 0 | .00071 | 0 | 0 | 0 | 1.60944 | 0 | .00115 | 0 | 0 | 0 |
| timidezza | 0 | 0 | 0 | 1 | 0 | 1 | 0 | 0 | 0 | .00056 | 0 | 1.60944 | 0 | 0 | 0 | .00090 | 0 |
| timor* | 3 | 1 | 0 | 1 | 1 | 6 | .00124 | .00071 | 0 | .00056 | .00055 | .22314 | .00028 | .00016 | 0 | .00012 | .00012 |
| tinder | 0 | 0 | 0 | 0 | 1 | 1 | 0 | 0 | 0 | 0 | .00055 | 1.60944 | 0 | 0 | 0 | 0 | .00089 |
| tocc* | 4 | 2 | 0 | 0 | 3 | 9 | .00165 | .00142 | 0 | 0 | .00166 | .51083 | .00084 | .00073 | 0 | 0 | .00085 |
| toy* | 1 | 4 | 11 | 163 | 10 | 189 | .00041 | .00285 | .01108 | .09121 | .00553 | 0 | 0 | 0 | 0 | 0 | 0 |
| tragico | 1 | 0 | 0 | 0 | 0 | 1 | .00041 | 0 | 0 | 0 | 0 | 1.60944 | .00066 | 0 | 0 | 0 | 0 |
| tranquill* | 8 | 0 | 3 | 0 | 7 | 18 | .00330 | 0 | .00302 | 0 | .00387 | .51083 | .00169 | 0 | .00154 | 0 | .00198 |
| trasgress* | 3 | 3 | 0 | 0 | 0 | 6 | .00124 | .00214 | 0 | 0 | 0 | .91629 | .00114 | .00196 | 0 | 0 | 0 |
| travestitismo | 0 | 1 | 0 | 0 | 0 | 1 | 0 | .00071 | 0 | 0 | 0 | 1.60944 | 0 | .00115 | 0 | 0 | 0 |
| travolgente | 1 | 0 | 0 | 0 | 0 | 1 | .00041 | 0 | 0 | 0 | 0 | 1.60944 | .00066 | 0 | 0 | 0 | 0 |
| trist* | 20 | 2 | 2 | 0 | 1 | 25 | .00826 | .00142 | .00201 | 0 | .00055 | .22314 | .00184 | .00032 | .00045 | 0 | .00012 |
| turbat* | 1 | 0 | 0 | 0 | 1 | 2 | .00041 | 0 | 0 | 0 | .00055 | .91629 | .00038 | 0 | 0 | 0 | .00051 |
| tv | 1 | 0 | 0 | 0 | 0 | 1 | .00041 | 0 | 0 | 0 | 0 | 1.60944 | .00066 | 0 | 0 | 0 | 0 |
| uomini | 0 | 1 | 0 | 0 | 1 | 2 | 0 | .00071 | 0 | 0 | .00055 | .91629 | 0 | .00065 | 0 | 0 | .00051 |
| utensili | 0 | 0 | 0 | 2 | 0 | 2 | 0 | 0 | 0 | .00112 | 0 | 1.60944 | 0 | 0 | 0 | .00180 | 0 |
| utero | 1 | 0 | 0 | 0 | 0 | 1 | .00041 | 0 | 0 | 0 | 0 | 1.60944 | .00066 | 0 | 0 | 0 | 0 |
| vaginal* | 0 | 0 | 1 | 1 | 1 | 3 | 0 | 0 | .00101 | .00056 | .00055 | .51083 | 0 | 0 | .00051 | .00029 | .00028 |
| varietà | 0 | 2 | 0 | 0 | 0 | 2 | 0 | .00142 | 0 | 0 | 0 | 1.60944 | 0 | .00229 | 0 | 0 | 0 |
| verbale | 1 | 0 | 0 | 0 | 1 | 2 | .00041 | 0 | 0 | 0 | .00055 | .91629 | .00038 | 0 | 0 | 0 | .00051 |
| vergin* | 1 | 0 | 0 | 0 | 1 | 2 | .00041 | 0 | 0 | 0 | .00055 | .91629 | .00038 | 0 | 0 | 0 | .00051 |
| vergogn* | 3 | 0 | 1 | 1 | 9 | 14 | .00124 | 0 | .00101 | .00056 | .00498 | .22314 | .00028 | 0 | .00022 | .00012 | .00111 |
| vestitini | 0 | 0 | 0 | 0 | 1 | 1 | 0 | 0 | 0 | 0 | .00055 | 1.60944 | 0 | 0 | 0 | 0 | .00089 |
| vibrator* | 0 | 1 | 2 | 13 | 0 | 16 | 0 | .00071 | .00201 | .00727 | 0 | .51083 | 0 | .00036 | .00103 | .00372 | 0 |
| video* | 9 | 4 | 11 | 63 | 10 | 97 | .00372 | .00285 | .01108 | .03525 | .00553 | 0 | 0 | 0 | 0 | 0 | 0 |
| violento | 2 | 0 | 0 | 0 | 0 | 2 | .00083 | 0 | 0 | 0 | 0 | 1.60944 | .00133 | 0 | 0 | 0 | 0 |
| virtual* | 11 | 0 | 2 | 5 | 3 | 21 | .00454 | 0 | .00201 | .00280 | .00166 | .22314 | .00101 | 0 | .00045 | .00062 | .00037 |
| vogli* | 30 | 23 | 15 | 3 | 11 | 82 | .01239 | .01638 | .01511 | .00168 | .00609 | 0 | 0 | 0 | 0 | 0 | 0 |
| vol* | 2 | 0 | 2 | 0 | 0 | 4 | .00083 | 0 | .00201 | 0 | 0 | .91629 | .00076 | 0 | .00185 | 0 | 0 |
| volgare | 0 | 0 | 0 | 0 | 1 | 1 | 0 | 0 | 0 | 0 | .00055 | 1.60944 | 0 | 0 | 0 | 0 | .00089 |
| volto | 1 | 0 | 0 | 0 | 0 | 1 | .00041 | 0 | 0 | 0 | 0 | 1.60944 | .00066 | 0 | 0 | 0 | 0 |
| web | 0 | 1 | 0 | 1 | 0 | 2 | 0 | .00071 | 0 | .00056 | 0 | .91629 | 0 | .00065 | 0 | .00051 | 0 |
| webcam | 0 | 0 | 1 | 6 | 0 | 7 | 0 | 0 | .00101 | .00336 | 0 | .91629 | 0 | 0 | .00092 | .00308 | 0 |
| webinar | 0 | 0 | 0 | 0 | 1 | 1 | 0 | 0 | 0 | 0 | .00055 | 1.60944 | 0 | 0 | 0 | 0 | .00089 |
| whatsapp | 0 | 0 | 0 | 2 | 0 | 2 | 0 | 0 | 0 | .00112 | 0 | 1.60944 | 0 | 0 | 0 | .00180 | 0 |
| xconfession | 0 | 0 | 0 | 1 | 0 | 1 | 0 | 0 | 0 | .00056 | 0 | 1.60944 | 0 | 0 | 0 | .00090 | 0 |
| yogiche | 0 | 0 | 0 | 1 | 0 | 1 | 0 | 0 | 0 | .00056 | 0 | 1.60944 | 0 | 0 | 0 | .00090 | 0 |
| youporn | 0 | 0 | 1 | 4 | 0 | 5 | 0 | 0 | .00101 | .00224 | 0 | .91629 | 0 | 0 | .00092 | .00205 | 0 |
| zon* | 1 | 1 | 0 | 0 | 2 | 4 | .00041 | .00071 | 0 | 0 | .00111 | .51083 | .00021 | .00036 | 0 | 0 | .00057 |
|  |  |  |  |  |  |  |  |  |  |  |  |  |  |  |  |  |  |
| **Total** | 2.421 | 1.404 | 993 | 1.787 | 1.807 |  |  |  |  |  |  |  |  |  |  |  |  |

Frequency information about words extracted from answers to open-ended questions are reported. In the first column. word stems are shown. Frequencies (F(1-5). total frequencies (F(total)). term frequencies (TF(1-5)). inverse document frequencies (IDF) and term-frequency-inverse document frequencies (TF-IDF(1-5)) related to the five context are reported for each term. The last row “Total” shows the measure of the length of each document, where the latter is considered as the total number of stems for each open-ended question multiplied by their frequency *(F(1-5)).*
