# Supplementary material for: The sexual experience of Italian adults during the COVID-19 lockdown
Source: PLoS One. 2022 May 5;17(5):e0268079. doi: 10.1371/journal.pone.0268079 (PMC9070892; doi:10.1371/journal.pone.0268079)
Supplement: S3 Table — Euclidean distance between the 7 terms with higher TF-IDF in the text corpus of answers to open-ended Question 2 are reported. The greater the Euclidean distance, the greater the distance/dissimilarity between items. (DOCX) [file pone.0268079.s003.docx]

**S3 Table. Euclidean Distance Matrix between Roots with higher TF-IDF in Question 2.**

|  | Dream* | See* | BDSM | Variety | Transgress* | Extreme* | Intens* |
| --- | --- | --- | --- | --- | --- | --- | --- |
| Dream* | 0 |  |  |  |  |  |  |
| See* | .001 | 0 |  |  |  |  |  |
| BDSM | .001 | 0 | 0 |  |  |  |  |
| Variety | .001 | 0 | 0 | 0 |  |  |  |
| Transgress* | .001 | 0 | 0 | 0 | 0 |  |  |
| Extreme* | .001 | 0 | 0 | 0 | 0 | 0 |  |
| Intens* | .002 | .001 | .001 | 0 | 0 | 0 | 0 |

Euclidean distance between the 7 terms with higher TF-IDF in the text corpus of answers to open-ended Question 2 are reported. The greater the Euclidean distance, the greater the distance/dissimilarity between items.
