# Supplementary material for: The sexual experience of Italian adults during the COVID-19 lockdown
Source: PLoS One. 2022 May 5;17(5):e0268079. doi: 10.1371/journal.pone.0268079 (PMC9070892; doi:10.1371/journal.pone.0268079)
Supplement: S4 Table — Euclidean distance between the 10 terms with higher TF-IDF in the text corpus of answers to open-ended Question 3 are reported. The greater the Euclidean distance, the greater the distance/dissimilarity between items. (DOCX) [file pone.0268079.s004.docx]

**S4 Table. Euclidean Distance Matrix between Roots with higher TF-IDF in Question 3.**

|  | Privacy | Shower | Loneliness | Fatigue* | Intens* | Relax* | Calm* | Date* | Movie* | Leisure |
| --- | --- | --- | --- | --- | --- | --- | --- | --- | --- | --- |
| Privacy | 0 |  |  |  |  |  |  |  |  |  |
| Shower | 0 | 0 |  |  |  |  |  |  |  |  |
| Loneliness | .001 | 0 | 0 |  |  |  |  |  |  |  |
| Fatigue* | .001 | 0 | 0 | 0 |  |  |  |  |  |  |
| Intens* | .001 | 0 | 0 | 0 | 0 |  |  |  |  |  |
| Relax* | .001 | 0 | 0 | 0 | 0 | 0 |  |  |  |  |
| Calm* | .001 | 0 | 0 | 0 | 0 | 0 | 0 |  |  |  |
| Date* | .001 | 0 | 0 | 0 | 0 | 0 | 0 | 0 |  |  |
| Movie* | .001 | 0 | 0 | 0 | 0 | 0 | 0 | 0 | 0 |  |
| Leisure | .001 | 0 | 0 | 0 | 0 | 0 | 0 | 0 | 0 | 0 |

Euclidean distance between the 10 terms with higher TF-IDF in the text corpus of answers to open-ended Question 3 are reported. The greater the Euclidean distance, the greater the distance/dissimilarity between items.
