# Supplementary material for: The sexual experience of Italian adults during the COVID-19 lockdown
Source: PLoS One. 2022 May 5;17(5):e0268079. doi: 10.1371/journal.pone.0268079 (PMC9070892; doi:10.1371/journal.pone.0268079)
Supplement: S5 Table — Euclidean distance between the 16 terms with higher TF-IDF in the text corpus of answers to open-ended Question 5 are reported. The greater the Euclidean distance, the greater the distance/dissimilarity between items. (DOCX) [file pone.0268079.s005.docx]

**S5 Table. Euclidean Distance Matrix between Roots with higher TF-IDF in Question 5.**

|  | Underst* | Know* | Emotion* | Foreplay | Bonding | Tenderness | Orientation | Love* | Affect* | Kiss* | Attract* | Grant* | Imperfect* | Patience | Unlock me | Dirty |
| --- | --- | --- | --- | --- | --- | --- | --- | --- | --- | --- | --- | --- | --- | --- | --- | --- |
| Underst* | 0 |  |  |  |  |  |  |  |  |  |  |  |  |  |  |  |
| Know* | 0 | 0 |  |  |  |  |  |  |  |  |  |  |  |  |  |  |
| Emotion* | 0 | 0 | 0 |  |  |  |  |  |  |  |  |  |  |  |  |  |
| Foreplay | 0 | 0 | 0 | 0 |  |  |  |  |  |  |  |  |  |  |  |  |
| Bonding | 0 | 0 | 0 | 0 | 0 |  |  |  |  |  |  |  |  |  |  |  |
| Tenderness | 0 | 0 | 0 | 0 | 0 | 0 |  |  |  |  |  |  |  |  |  |  |
| Orientation | 0 | 0 | 0 | 0 | 0 | 0 | 0 |  |  |  |  |  |  |  |  |  |
| Love* | 0 | 0 | 0 | 0 | 0 | 0 | 0 | 0 |  |  |  |  |  |  |  |  |
| Affect* | .001 | 0 | 0 | 0 | 0 | 0 | 0 | 0 | 0 |  |  |  |  |  |  |  |
| Kiss* | .001 | .001 | 0 | 0 | 0 | 0 | 0 | 0 | 0 | 0 |  |  |  |  |  |  |
| Attract* | .001 | .001 | 0 | 0 | 0 | 0 | 0 | 0 | 0 | 0 | 0 |  |  |  |  |  |
| Grant* | .001 | .001 | .001 | .001 | .001 | .001 | 0 | 0 | 0 | 0 | 0 | 0 |  |  |  |  |
| Imperfect* | .001 | .001 | .001 | .001 | .001 | .001 | 0 | 0 | 0 | 0 | 0 | 0 | 0 |  |  |  |
| Patience | .001 | .001 | .001 | .001 | .001 | .001 | 0 | 0 | 0 | 0 | 0 | 0 | 0 | 0 |  |  |
| Unlock me | .001 | .001 | .001 | .001 | .001 | .001 | 0 | 0 | 0 | 0 | 0 | 0 | 0 | 0 | 0 |  |
| Dirty | .001 | .001 | .001 | .001 | .001 | .001 | 0 | 0 | 0 | 0 | 0 | 0 | 0 | 0 | 0 | 0 |

Euclidean distance between the 16 terms with higher TF-IDF in the text corpus of answers to open-ended Question 5 are reported. The greater the Euclidean distance, the greater the distance/dissimilarity between items.
