# Supplementary material for: The sexual experience of Italian adults during the COVID-19 lockdown
Source: PLoS One. 2022 May 5;17(5):e0268079. doi: 10.1371/journal.pone.0268079 (PMC9070892; doi:10.1371/journal.pone.0268079)
Supplement: S6 Table — Summary of cluster solutions. At each stage, the cases with the smallest Euclidean distance are combined; the coefficients indicating cluster heterogeneity change when a case is combined with the cluster. The solution before the largest gap in the coefficient indicates the best cluster solution (Stage 28). (DOCX) [file pone.0268079.s006.docx]

**S6 Table. Agglomeration Schedule for Complete Linkage of roots with higher TF-IDF in Question 1.**

| Stage | Cluster Combined | | Coefficients | Stage Cluster First Appears | | Next stage |
| --- | --- | --- | --- | --- | --- | --- |
|  | Cluster 1 | Cluster 2 |  | Cluster 1 | Cluster 2 |  |
| 1 | Reinvent* | Relief | 0 | 0 | 0 | 2 |
| 2 | Surrender* | Reinvent* | 0 | 0 | 1 | 4 |
| 3 | Insatiable | Argu* | 0 | 0 | 0 | 4 |
| 4 | Surrender* | Insatiable | 0 | 2 | 3 | 6 |
| 5 | Impotence | Unwanted | 0 | 0 | 0 | 6 |
| 6 | Surrender* | Impotence | 0 | 4 | 5 | 7 |
| 7 | Surrender* | Contrasting | 0 | 6 | 0 | 19 |
| 8 | Affect* | Abstinence | 0 | 0 | 0 | 18 |
| 9 | Frustr* | Sad* | 0 | 0 | 0 | 20 |
| 10 | Happy* | Melancholy* | 0 | 0 | 0 | 15 |
| 11 | Confused | Innovativ* | 0 | 0 | 0 | 12 |
| 12 | Comfort* | Confused | 0 | 0 | 11 | 17 |
| 13 | Repress* | Listless* | 0 | 0 | 0 | 14 |
| 14 | Asexual* | Repress* | 0 | 0 | 13 | 26 |
| 15 | Loneliness | Happy* | 0 | 0 | 10 | 20 |
| 16 | Calm* | Relax* | 0 | 0 | 0 | 24 |
| 17 | Anxi* | Comfort* | 0 | 0 | 12 | 23 |
| 18 | See* | Affect* | 0 | 0 | 8 | 21 |
| 19 | Satisf* | Surrender* | 0 | 0 | 7 | 25 |
| 20 | Loneliness | Frustr* | 0 | 15 | 9 | 23 |
| 21 | Passion* | See* | 0 | 0 | 18 | 24 |
| 22 | Intens* | Nervous* | 0 | 0 | 0 | 27 |
| 23 | Anxi* | Loneliness | 0 | 17 | 20 | 27 |
| 24 | Calm* | Passion* | 0 | 16 | 21 | 25 |
| 25 | Calm* | Satisf* | 0 | 24 | 19 | 28 |
| 26 | Depress* | Asexual* | 0 | 0 | 14 | 29 |
| 27 | Intens* | Anxi* | 0 | 22 | 23 | 28 |
| 28 | Intens* | Calm* | 0 | 27 | 25 | 30 |
| 29 | Suffer* | Depress* | .001 | 0 | 26 | 30 |
| 30 | Suffer* | Intens* | .001 | 29 | 28 | 0 |

Summary of cluster solutions. At each stage, the cases with the smallest Euclidean distance are combined; the coefficients indicating cluster heterogeneity change when a case is combined with the cluster. The solution before the largest gap in the coefficient indicates the best cluster solution (Stage 28).

Summary of cluster solutions. At each stage, the cases with the smallest Euclidean distance are combined; the coefficients indicating cluster heterogeneity change when a case is combined with the cluster. The solution before the largest gap in the coefficient indicates the best cluster solution (Stage 28).
