# Supplementary material for: The sexual experience of Italian adults during the COVID-19 lockdown
Source: PLoS One. 2022 May 5;17(5):e0268079. doi: 10.1371/journal.pone.0268079 (PMC9070892; doi:10.1371/journal.pone.0268079)
Supplement: S7 Table — Summary of cluster solutions. At each stage, the cases with the smallest Euclidean distance are combined; the coefficients indicating cluster heterogeneity change when a case is combined with the cluster. The solution before the largest gap in the coefficient indicates the best cluster solution (Stage 4). (DOCX) [file pone.0268079.s007.docx]

**S7 Table. Agglomeration Schedule for Complete Linkage of roots with higher TF-IDF in Question 2.**

| Stage | Cluster Combined | | Coefficients | Stage Cluster First Appears | | Next stage |
| --- | --- | --- | --- | --- | --- | --- |
|  | Cluster 1 | Cluster 2 |  | Cluster 1 | Cluster 2 |  |
| 1 | Transgress* | Extreme* | 0 | 0 | 0 | 4 |
| 2 | See* | BDSM | 0 | 0 | 0 | 3 |
| 3 | See* | Variety | 0 | 2 | 0 | 5 |
| 4 | Transgress* | Intens* | 0 | 1 | 0 | 5 |
| 5 | See* | Transgress* | .001 | 3 | 4 | 6 |
| 6 | Dream* | See* | .002 | 0 | 5 | 0 |

Summary of cluster solutions. At each stage, the cases with the smallest Euclidean distance are combined; the coefficients indicating cluster heterogeneity change when a case is combined with the cluster. The solution before the largest gap in the coefficient indicates the best cluster solution (Stage 4).
