# Supplementary material for: The sexual experience of Italian adults during the COVID-19 lockdown
Source: PLoS One. 2022 May 5;17(5):e0268079. doi: 10.1371/journal.pone.0268079 (PMC9070892; doi:10.1371/journal.pone.0268079)
Supplement: S10 Table — (DOCX) [file pone.0268079.s010.docx]

**S10 Table. Frequencies of participants’ responses to sociodemographic questionnaire for binary gender (male/female) derived from responses to the question “sex as assigned at birth.”**

|  | N  (respondents) | | Male sex | | Female  sex | |
| --- | --- | --- | --- | --- | --- | --- |
|  | N  (%) | Mean (SD) | n  (%) | Mean (SD) | n  (%) | Mean (SD) |
| 1. Age: | 465  (100) | 29.26  (1.37) | 99  (21.3) | 33.01  (12.18) | 366  (78.7) | 28.25  (9.59) |
| 2. Gender: | 465  (100) |  | 99  (21.3) |  | 366  (78.7) |  |
| Male | 98  (21.1) |  | 98  (21.1) |  | 0 |  |
| Female | 354  (76.1) |  | 0 |  | 354  (76.1) |  |
| Androgyne | 1  (.2) |  | 0 |  | 1  (.2) |  |
| Bigender | 2  (.4) |  | 0 |  | 2  (.4) |  |
| Cis woman | 2  (.4) |  | 0 |  | 2  (.4) |  |
| Cisgender woman | 2  (.4) |  | 0 |  | 2  (.4) |  |
| Female to male | 1  (.2) |  | 0 |  | 1  (.2) |  |
| Gender fluid | 2  (.4) |  | 0 |  | 2  (.4) |  |
| Gender queer | 1  (.2) |  | 1  (.2) |  | 0 |  |
| Nonbinary | 2  (.4) |  | 0 |  | 2  (.4) |  |
| 4.Sexual Orientation: | 465  (100) |  | 99  (21.3) |  | 366  (78.7) |  |
| Hetero | 333  (71.6) |  | 75  (16.1) |  | 258  (55.5) |  |
| Hetero + Homo | 77  (16.6) |  | 8  (1.7) |  | 69  (14.8) |  |
| Hetero ++ Homo | 12  (2.6) |  | 1  (.2) |  | 11  (2.4) |  |
| Bisexual | 16  (3.4) |  | 1  (.2) |  | 15  (3.2) |  |
| Homo + Hetero | 3  (.6) |  | 2  (.4) |  | 1  (.2) |  |
| Homo ++ Hetero | 13  (2.8) |  | 6  (1.3) |  | 7  (1.5) |  |
| Homo | 11  (2.4) |  | 6  (1.3) |  | 5  (1.1) |  |
| 5. Partner: | 465  (100) |  | 99  (21.3) |  | 366  (78.7) |  |
| Yes | 332  (71.4) |  | 63  (13.5) |  | 269  (57.8) |  |
| No | 133  (28.6) |  | 36  (7.7) |  | 97  (2.8) |  |
| 6. Civil Status: | 464  (100) |  | 99  (21.3) |  | 365  (78.7) |  |
| Single | 341  (73.5) |  | 69  (14.9) |  | 272  (58.6) |  |
| Religious Marriage | 27  (5.8) |  | 6  (1.3) |  | 21  (4.5) |  |
| Civil Marriage | 16  (3.4) |  | 8  (1.7) |  | 8  (1.7) |  |
| Separate/Divorced | 19  (4.1) |  | 3  (.6) |  | 16  (3.4) |  |
| Cohabitating | 58  (12.5) |  | 12  (2.6) |  | 46  (9.9) |  |
| Widow | 3  (.6) |  | 1  (.2) |  | 2  (.4) |  |
| 7. With whom the lockdown period was experienced: | 465  (100) |  | 99  (21.3) |  | 366  (78.7) |  |
| Family (e.g., parents) | 231  (49.7) |  | 46  (9.9) |  | 185  (39.8) |  |
| Partner* | 76  (16.3) |  | 20  (4.3) |  | 56  (12.0) |  |
| Children | 15  (3.3) |  | 4  (.9) |  | 11  (2.4) |  |
| Other Tenants/Roommates | 26  (5.6) |  | 3  (.6) |  | 23  (4.9) |  |
| Alone | 43  (9.2) |  | 12  (2.6) |  | 31  (6.6) |  |
| Isolation Center | 0 |  | 0 |  | 0 |  |
| Other | 74  (15.9) |  | 14  (3.0) |  | 60  (12.9) |  |
| 8. Children you lived with: | 465  (100) |  | 99  (21.3) |  | 366  (78.7) |  |
| None | 403  (86.6) |  | 85  (18.3) |  | 318  (68.4) |  |
| One | 30  (6.5) |  | 7  (1.5) |  | 23  (4.9) |  |
| Two | 24  (5.2) |  | 6  (1.3) |  | 18  (3.9) |  |
| Three | 6  (1.3) |  | 0 |  | 6  (1.3) |  |
| More than three | 2  (.4) |  | 1  (.2) |  | 1  (.2) |  |
| 9. Level of Education: | 464  (100) |  | 99  (21.3) |  | 365  (78.7) |  |
| Primary School Diploma (Elementary) | 0 |  | 0 |  | 0 |  |
| Middle School Diploma | 9  (1.9) |  | 3  (.6) |  | 6  (1.3) |  |
| Few Years of Secondary School (Higher) | 3  (.6) |  | 2  (.4) |  | 1  (.2) |  |
| Secondary School Diploma (Higher) | 122  (26.3) |  | 32  (6.9) |  | 90  (19.4) |  |
| Bachelor’s Degree | 165  (35.6) |  | 27  (5.8) |  | 138  (29.7) |  |
| Master’s Degree | 114  (24.6) |  | 24  (5.2) |  | 90  (19.4) |  |
| Post Graduate Master/PhD | 51  (11.0) |  | 11  (2.4) |  | 40  (8.6) |  |
| 1. Job: | 463  (100) |  | 98  (21.2) |  | 365  (78.8) |  |
| Professional | 70  (15.1) |  | 21  (4.5) |  | 49  (1.6) |  |
| Merchant | 6  (1.3) |  | 2  (.4) |  | 4  (.9) |  |
| Healthcare Worker | 32  (6.9) |  | 7  (1.5) |  | 25  (5.4) |  |
| Employee | 49  (1.6) |  | 12  (2.6) |  | 37  (8) |  |
| Worker | 9  (1.9) |  | 4  (.9) |  | 5  (1.1) |  |
| Teacher | 24  (5.2) |  | 5  (1.1) |  | 19  (4.1) |  |
| Student | 186  (4.2) |  | 27  (5.8) |  | 159  (34.3) |  |
| Retiree | 1  (.2) |  | 1  (.2) |  | 0 |  |
| Houseman/Housewife | 5  (1.1) |  | 0 |  | 5  (1.1) |  |
| Unemployed | 36  (7.8) |  | 6  (1.3) |  | 30  (6.5) |  |
| Other | 45  (9.7) |  | 13  (2.8) |  | 32  (6.9) |  |
| 11. Paid work during quarantine: | 465  (100) |  | 99  (21.3) |  | 366  (78.7) |  |
| Yes | 151  (32.5) |  | 44  (9.5) |  | 107  (23.0) |  |
| No | 314  (67.5) |  | 55  (11.8) |  | 259  (55.7) |  |
| 12. Work mode: | 149  (100) |  | 43  (28.9) |  | 366  (71.1) |  |
| Workplace | 55  (36.9) |  | 20  (13.4) |  | 35  (23.5) |  |
| Smart homework | 94  (63.1) |  | 23  (15.4) |  | 71  (47.7) |  |
| 13. Italian province where you lived: | 465  (100) |  | 99  (21.3) |  | 366  (78.7) |  |
| Rome | 37  (8.0) |  | 14  (3.0) |  | 23  (5.0) |  |
| Perugia | 195  (41.9) |  | 34  (7.3) |  | 161  (34.6) |  |
| Milan | 10  (2.2) |  | 4  (.9) |  | 6  (1.3) |  |
| Terni | 37  (7.9) |  | 14  (3.0) |  | 23  (4.9) |  |
| Other | 186  (4.0) |  | 33  (7.1) |  | 153  (32.9) |  |
| 14. Political Orientation: | 465  (100) |  | 99  (21.3) |  | 366  (78.7) |  |
| Right Wing | 33  (7.1) |  | 9  (1.9) |  | 24  (5.2) |  |
| Centre-Right Wing | 1  (.2) |  | 0 |  | 1  (.2) |  |
| Centre | 44  (9.5) |  | 14  (3.0) |  | 30  (6.5) |  |
| Centre-Left Wing | 67  (14.4) |  | 16  (3.4) |  | 51  (11.0) |  |
| Left Wing | 126  (27.1) |  | 23  (4.9) |  | 103  (22.2) |  |
| None | 110  (23.7) |  | 22  (4.7) |  | 88  (18.9) |  |
| I don’t know | 84  (18.0) |  | 15  (3.2) |  | 69  (14.8) |  |
| 15. Religion: | 465  (100) |  | 99  (21.3) |  | 366  (78.7) |  |
| Catholic Christian | 206  (44.3) |  | 46  (9.9) |  | 160  (34.4) |  |
| Orthodox Christian | 2  (.4) |  | 0 |  | 2  (.4) |  |
| Evangelical/Reformed Christian | 1  (.2) |  | 0 |  | 1  (.2) |  |
| Jewish | 0 |  | 0 |  | 0 |  |
| Islamic | 0 |  | 0 |  | 0 |  |
| Buddhist | 8  (1.7) |  | 1  (.2) |  | 7  (1.5) |  |
| No religion | 214  (46.0) |  | 43  (9.2) |  | 171  (36.8) |  |
| I don’t know | 34  (7.3) |  | 9  (1.9) |  | 25  (5.4) |  |
| 16. Respondent’s Disability: | 465  (100) |  | 99  (21.3) |  | 366  (78.7) |  |
| Yes | 26  (5.6) |  | 7  (1.5) |  | 19  (4.1) |  |
| No | 439  (94.4) |  | 92  (19.8) |  | 347  (74.6) |  |
| 17. Type of Respondent’s Disability: | 465  (100) |  | 99  (21.3) |  | 366  (78.7) |  |
| Invisible | 21  (4.5) |  | 7  (1.5) |  | 14  (3.0) |  |
| Visible | 1  (.2) |  | 0 |  | 1  (.2) |  |
| Sensory | 3  (.6) |  | 0 |  | 3  (.6) |  |
| (No Disability) | 440  (94.6) |  | 92  (19.8) |  | 348  (74.8) |  |
| 18. Children’s Disability: | 465  (100) |  | 99  (21.3) |  | 366  (78.7) |  |
| Yes | 6  (1.3) |  | 0 |  | 6  (1.3) |  |
| No | 459  (98.7) |  | 99  (21.3) |  | 360  (77.4) |  |
| 19. Type of Children’s Disability: | 464  (100) |  | 99  (21.3) |  | 365  (78.7) |  |
| Invisible | 3  (.6) |  | 0 |  | 3  (.6) |  |
| Visible | 2  (.4) |  | 0 |  | 2  (.4) |  |
| Sensory | 0 |  | 0 |  | 0 |  |
| (No Disability) | 459  (98.9) |  | 99  (21.3) |  | 360  (77.6) |  |
| 2. Was the romantic relationship maintained during the lockdown? | 465  (100) |  | 99  (21.3) |  | 366  (78.7) |  |
| Yes. long-distance | 180  (38.7) |  | 33  (7.1) |  | 147  (31.6) |  |
| Yes. cohabitating | 119  (25.6) |  | 27  (5.8) |  | 92  (19.8) |  |
| No. broke down | 16  (3.4) |  | 5  (1.1) |  | 11  (2.4) |  |
| Single | 119  (25.6) |  | 30  (6.5) |  | 89  (19.1) |  |
| Other | 31  (6.7) |  | 4  (.9) |  | 27  (5.8) |  |
| 21. Were there any changes in your sex life during the lockdown? | 355  (100) |  | 73  (2.6) |  | 282  (79.4) |  |
| No. long-distance | 109  (3.7) |  | 24  (6.8) |  | 85  (23.9) |  |
| No. lived together | 62  (17.5) |  | 15  (4.2) |  | 47  (13.2) |  |
| Yes. for the better (long-distance) | 63  (17.7) |  | 12  (3.4) |  | 51  (14.4) |  |
| Yes. for the better (lived together) | 47  (13.2) |  | 8  (2.2) |  | 39  (11) |  |
| Yes. for the worse (long-distance) | 47  (13.2) |  | 5  (1.4) |  | 42  (11.8) |  |
| Yes. for the worse (lived together) | 27  (7.6) |  | 9  (2.5) |  | 18  (5.1) |  |
| 22. Virus exposure: | 464  (100) |  | 99  (21.3) |  | 365  (78.7) |  |
| None (no test) | 395  (85.1) |  | 82  (17.7) |  | 313  (67.4) |  |
| None (with test) | 23  (5) |  | 6  (1.3) |  | 17  (3.7) |  |
| Exposed (no test) | 34  (7.3) |  | 8  (1.7) |  | 26  (5.6) |  |
| Lived/worked with infected | 9  (1.9) |  | 3  (.6) |  | 6  (1.3) |  |
| COVID-19 positive | 0 |  | 0 |  | 0 |  |
| I don’t want to answer | 3  (.6) |  | 0 |  | 3  (.6) |  |
