# Supplementary material for: The sexual experience of Italian adults during the COVID-19 lockdown
Source: PLoS One. 2022 May 5;17(5):e0268079. doi: 10.1371/journal.pone.0268079 (PMC9070892; doi:10.1371/journal.pone.0268079)
Supplement: Attachment — Submitted filename: Review_The sexual experience of Italian adults during the COVID-19 lockdown.pdf [file pone.0268079.s011.pdf]

The sexual experience of Italian adults during the COVID-19 lockdown

In this paper, the authors addressed the critical issues about the effect on sexual health and behavior due to the lockdown period experienced by 465 Italian participants. Nowadays, the topic is of great interest as the Covid-19 emergency has forced people to stay in quarantine, impacting their physical, psychological, and, no doubt, sexual health. I think this work offers valuable insight into this critical issue.

To investigate the sexual health and behaviors of Italian adults during the lockdown period, authors used three main instruments:

1. A Sociodemographic and Behavioral e-Questionnaire During Quarantine;
2. The Sexual Modes Questionnaire (SMQ) – Nonbinary Form: a closed-ended self-report questionnaire (five-point Likert-type scale) to assess the interactions between cognition, emotion, and sexual response.
3. An Open-ended e-Questionnaire on Sexual Experience. Here, participants were invited to respond to five open-ended questions with a maximum of 280 characters.

In the analysis of this last instrument, the TF-IDF index was used to extract the weight of some salient words employed by respondents to answer the five open-ended questions. I will focus precisely on this type of analysis as some points need to be clarified.

<u>Main points:</u>

1) After that participant responded to the five open-ended questions with a maximum of 280 characters, the authors extracted some relevant words to the questions asked and the study. Subsequentially, they isolated a "stoplist,": a set of empty words (articles, pronouns, conjunctions, etc.). Thus, a total number of 1.313 words (not belonging to this stoplist) were then considered for further analysis.
   On page 19, line 278, the authors wrote: *"to facilitate the analysis, the 1,313 words not belonging to the stoplist were associated with each other based on the root of each word (i.e., the part not subject to variation, which contains the fundamental meaning of the word), obtaining a final list of 607 roots S1 Table."* Confirming this, **the authors reported in the S1 Table description: "*Roots (N= 607)*". Despite that, in the S1 Table, I counted a little over 300 words, not 607.**

That being said, it's not clear for me if this is a simple typing error or if further operations to the original list of 607 roots were carried out and not reported in the main text.

In addition, it is essential to point out that the **lemmatization procedure** from which the authors then derive what they have called 'root words' **is a standard procedure that has not been mentioned**. Therefore, I recommend the authors provide the correct mention of the methodology adopted.

It is essential, in my opinion, to clarify this point since the total number of root words is indispensable for estimating the TF-IDF index.

2) After this first operation, the authors used the TF-IDF index to calculate the weight of each root within what they call the "5 contexts," i.e., the five questions that were asked to the participants in the questionnaire defined "Open-ended e-Questionnaire on Sexual Experience."

From my point of view, **the computation of the TF-IDF index is not well explained in the main text**; I suppose that an inexperienced reader who is not familiar with the formula will not fully understand its meaning. I would suggest the authors add the TF-IDF index formula and clarify what is referred to as TF and what is referred to as IDF. For example:

"… given a collection *C* of documents *d*, the TF-IDF value for each term *t* in a document $d \in C$ is calculated as:

$$\text{TF-IDF}(t,d,C) = \text{TF}(d,t) * \text{IDF}(t,C),$$

where TF (*d*, *t*) indicates the number of times a target term *t* appears in document *d*, and IDF is equal to $log(N/n)$, where *N* indicates the number of documents in *C* and *n* is the number of documents where *t* is used. In this study case, the TF part of the formula, the collection *C* is the total number of documents (*C* =…), the term *t* corresponds to each of the roots (e.g., "abbracc*"), and the document *d* is equal to each one of the five questions belonging to the collection *C*. Concerning the IDF formula instead, *N* is equal to etc..."

If this is not clarified, I think it is not easy to understand how the TF-IDF was calculated; in fact, based only on what the authors reported, there are some points in the calculation of the TF-IDF index that are not clear to me:

3) Starting from the TF, if I correctly understood, in your case, TF corresponds to the number of times a root *t* appears in document *d*, i.e., in each one of the five questions taken separately. For example, as reported in the S1 Table, if the root "abbracci*" frequency is equal to 2 in question number 1, it means that (taking the answers of all participants) this root was written two times in the participants text answers.

   Therefore, in this case, since no other operation is described in the manuscript, the TF is expected to be a whole number, as it is a frequency index. Nevertheless, in table S1, the TF corresponds to the number 0.00037. However, given that the TF should be divided by the length of the document itself (to avoid favoring longer documents), does the TF correspond to a decimal number because the frequency (e.g., F=2) has been divided by the length of the document text?

   I can guess from line 289 ("its frequency in a <u>specific context of use</u>") that it may have been divided by the total number of words in the document. Is this correct? If so, however, the length of each document is not clear since the authors extracted some words (e.g., the ones in the "stoplist"). **Could the authors please explain the TF calculation they have made?**

4) **Turning to the calculation of the IDF, again, in my opinion, the authors did not provide enough information.** The IDF is calculated as the *log(N/n)*. Since *N* indicates the total number of documents in *C* and *n* is the number of documents where *t* is used, could the authors specify what they have considered *N* and what they have considered *n*?

   From my point of view, in the case of the root "abbracci*," *n* should be equal to 3 since this term appears in 3 of the five documents (questions 1, 2, and 5). While it is not clear which value authors used to *N*. Following their reasoning, it should be 5, as the total number of the questions is five; however, if we apply these values, the IDF results are not the same reported in the Table S1.

To summarise the above, the procedure and methodology of useful words extraction and lemmatization are not well described and make way for interpretation by the reader. Moreover, I also recommend the authors provide the correct mention of the methodology adopted.

Furthermore, both TF and IDF calculations are standard procedures that, in my opinion, must be well-described step by step. As presented now, it is unclear which components of the text were used to calculate the TF-IDF, thus not allowing the reproducibility of the results.

Minor points:

One the last clarification on TF-IDF: in line 410, authors wrote: "After applying the TF-IDF [31] weight function, where **"DF = document frequency"** was considered the **occurrence of a root in each of the five open-ended questions."** Do the authors refer to the TF?

Moreover, in two parts of the manuscript (lines 906, 907), the 'TF' is translated as *text* frequency instead of *term* frequency.

In the online Excel table with the raw data provided by the authors, the last column collects **some emails from participants in which their first and last names are visible**. I strongly recommend that the authors make the data anonymous so that participants' identities cannot be traced.

From a practical point of view, the English language is clear and of sufficient quality to be understood. I remind authors to **add the page number at the bottom of the pages**.

The title and keywords encapsulate the paper's content, and the major innovative point of this study is clearly explained and even included in the abstract. The introduction section is also well done; the authors provided a rich overview of current literature on the field, including references to recent and past studies.

The tables and figures are well done, although **I would make Table S10 in the same format as the others so that they are all similar.** I also appreciated the paper's structure and the organization of the paragraphs with their respective titles. The manuscript structure and format respect the PlosOne journal criteria.
