# Supplementary material for: The sexual experience of Italian adults during the COVID-19 lockdown
Source: PLoS One. 2022 May 5;17(5):e0268079. doi: 10.1371/journal.pone.0268079 (PMC9070892; doi:10.1371/journal.pone.0268079)
Supplement: Attachment — Submitted filename: Federici et al_2021_The Sexual Experience_RtoR_REV2.docx [file pone.0268079.s013.docx]

**Response to reviewers’ comments**

Below we provide a point-by-point response to the reviewers’ comments and suggestions.

- **R2** = Reviewer 2 comment; **RP2** = Response to Reviewer 2 comment
- **R3** = Reviewer 3 comment; **RP3** = Response to Reviewer 3 comment

**Reviewer 2**

1. **R2**: “I will quote my previous remarks for an easier understanding. 3R2: My major concerns remain the nature of the five questions, many of which encompassed more than one question and suggest a possible answer. For example question 2: “How did you do with your sexual desire and arousal? Have you noticed a change in your erotic fantasies?”: the second question suggests that there was a change in sexual fantasies; same for question 3; question 4 asks two different questions while question 5 three different ones. The authors should make it clear in the Discussion section that this is a limitation of their study that may have led to not completely frank answers.”
   - **RP2**: According to your request, we have added your criticism in the Discussion section as follows:

“During the review process of the present study, one of the reviewers pointed out to us a limitation regarding the wording of the Open-ended e-Questionnaire on Sexual Experience. Some of the five questions included more than one question, which might suggest a possible answer. For example, question 2: “How did you do with your sexual desire and arousal? Have you noticed a change in your erotic fantasies?”: The second question might suggest that there was a change in sexual fantasies. This might have led respondents to not completely frank answers, according to the reviewer.”

2. **R2**: “4R2: I found no tracks of any correction in the Introduction section, but for the change of “Predictions” into “hypotheses”. So my doubts are just the same as before. Please change the sentence about international publications: as it stands now it is misleading.”
   - **RP2**: It is not our intention to make a trivial facade change, a simple maquillage just to meet your requirements. We are sincerely sorry if our correction gave you that impression. We have consulted with other colleagues and searched the relevant scientific literature for what exactly you are asking us to change. But your request continues to be unclear to us.

Anyway, we tried to make some changes to address your comment. We are aware that these changes are minimal. But it is not our intention in any way to minimize your request. The current changes and the original text are based on the following methodological assumptions:

(i) Our study design does not allow us to control for all intervening variables and establish or hypothesize a causal direction between independent and dependent variables;
(ii) Our study design hypothesizes a positive directional relationship between the variables/factors;
(iii) Our hypotheses are uttered in declarative form (declarative hypothesis vs. null form) because we make a positive statement about the outcome of the study.

Having said that, we made the following changes. Before the subsection began, we included a sentence as follows:

“We next present our expectations about the association between the lockdown condition and sexual health and behaviors of Italian adults.”

We titled the subsection “Expectations” as suggested in scientific studies such as [https://doi.org/10.1509/jm.15.0340](https://doi.org/10.1509%2Fjm.15.0340).

We changed the first sentence of the subsection as follows:

“Our expectations assume that the lockdown situation experienced in the first months of the pandemic may have altered various aspects of Italians’ sexuality, due to the changes in daily life imposed by the current COVID-19 outbreak.”

3. **R2**: “6R2: I find your answer not appropriate: Sex assigned at birth is not relevant in a psychological study: you should rerun your analysis considering gender identity, and limiting them to persons that identify themself as male or female since the other groups were too little and cannot be encompassed in the sample unless losing data clarity.
   - **RP2**: We would like to sincerely thank you for your careful review of our manuscript and for all the time you devoted to it. It has always been our intention to take your comments seriously, welcoming them as an opportunity to improve our study. However, we were stunned by your statement “Sex assigned at birth is not relevant in a psychological study.” One can certainly believe that the earth is flat, but it is not up to us to give justification as to why it is round. Please provide us with references from the scientific community stating that it is unjustified in psychological studies to split a sample by sex. If we have missed statements and studies from the American Psychological Association, American Psychiatric Association, or even PlosOne’s policy we are willing to reconsider our data analyses. Otherwise, we like every other study published in international scientific Journals, we do not have to justify why we divided the sample by sex, while we should justify why we should divide it by gender.
4. **R2**: “6R2: Furthermore, it is not important how other people or even you are carrying out other research, in this one you used the old Kinsey scale, quoting it, and that does not encompass asexuality. Please quote that in the study limitation”
   - **RP2**: We have added this limitation at the end of the Discussion as follows:

“Another limitation of the study concerns the use of the Kinsey scale to identify sexual orientation. We used the (old) seven-item version which did not include to account for asexuality. This may have limited some respondents in finding appropriate identification regarding their sexual orientation.”

5. **R2**: “9R2: you just deleted that part from the paper. I know what quintiles are and their use: I don’t need a lesson about that. If the data are not normally distributed quintiles are not appropriate for determining a cut-off.”
   - **RP2**: It was not our intention to teach you anything or rant by giving you a lesson on quantiles. If we have given you this impression, and we apologize, it is only because we are trying to justify to you our methodological choices. According to our scientific and statistical knowledge, we disagree with your claim that “If the data are not normally distributed quintiles are not appropriate for determining a cut-off.” Please give us references to scientific publications of statistics that support your comment. According to our knowledge of statistics and the most famous scientific manuals and Journal articles, we believe that we have used a correct data analysis. Now below, we do not want to teach you a lesson, but wish to base our methodological choices on scientific evidence. For example, Kenney and Keeprin (1966) – cited on Google Scholar almost 2000 times and by some authors who themselves have more than 8000 citations – explain that quantiles, and specifically quartiles, are used not only to describe but even to calculate a skewness, in the same way as the more famous Pearson’s Skewness does. This formula is called Bowley skewness or Galton skewness. As more recently mentioned by Jones et al. (2011), “quantile-based measures of kurtosis and their interaction with skewness-inducing transformations, identifying classes of transformations that leave kurtosis measures invariant.” (p. 89). This makes us argue not only that using quartiles a skewness is a routine procedure, but also that it has important advantages for our study.
6. **R2**: “11R2: I still think that t-test and Anova on single items are not appropriate tests. Furthermore, if this is allowed (but a statistician must answer to that) you should use the Bonferroni correction for the p-value: .05 x items N.”
   - **RP2**: A statistician (Reviewer #3) has already declared, as you can check on Editorial Manager, that the manuscript is technically sound, and the statistical analysis has been performed appropriately and rigorously.

However, we reiterate what we have already explained in the previous “Response to reviewers’ comments”. We have no normative data for SMQ. The original English version of SMQ was composed of two forms: the male form with 30 items divided into 5 factors (failure anticipation thoughts, erection concerns thoughts, age- and body-related thoughts, negative thoughts toward sex and lack of erotic thoughts) and the female form with 33 items divided into 6 factors (sexual abuse thoughts, failure and disengagement thoughts, partner’s lack of erotic thoughts) and the female version with 33 items divided into 6 factors (sexual abuse thoughts, failure and disengagement thoughts, partner’s lack of affection, sexual passivity, lack of erotic thoughts and low self-body image thoughts). For the present study, we created a new “non-binary version” by combining the two versions that not only have a different number of items but also different factors. Understanding how each item behaves is extremely important to us since we do not have validated data on the factorial structure on this new Italian version.

About the Bonferroni correction, we use it by default how you can see from our output. We apologize for not specifying it in the article. We have added ita the line 357 of the main text as follows: “Where appropriate, Bonferroni’s corrections have been applied.”

7. **R2**: “13R2: I appreciate the changes.”
   - **RP2**: Thank you for your appreciation.
8. **R2**: “14R2: In lines 617-619 you made an interesting hypothesis. Nevertheless, I find it very difficult to follow the discussion since there are no means reported in the paper and I don’t know the direction of the significant difference: if the mean difference is positive it means that persons with disabilities had a higher mean than persons without disabilities? I would appreciate it if you can put some examples from your data in this part of the Discussion section that emphasized this aspect.”
   - **RP2**: We thank you very much for this comment that give as the chance to improve the quality of our study by making it clearer. We have amended Tables 2 and 3 by adding a column with “Mean Differences” between participants with and without disability. In addition, in the Discussion section we have included references to those tables in parentheses when discussing the effect of disability on sexual behavior (lines 584-585).

**Reviewer 3**

9. **R3**: “I have read the authors’ responses to my comments. The authors fixed what I had pointed out and clarified some minor points I had asked them to justify. Above all, they removed the participants’ emails from the Excel file containing the online research data to guarantee the anonymity of the participants. Turning to the major points, the authors correctly updated the table by adding the missing elements from the list of 607 words they had indicated in the text. They also updated the TF-IDF values compared to the previous table uploaded. The authors also provided more detailed information about the stemming process regarding the adopted procedure:

“Then, to facilitate the analysis, the stemming procedure was used to reduce inflected (or derived) words to their word stem (i.e., the main part of a word that stays the same when endings are added to it). Whole words (i.e., not the stem) were maintained when present only once in the list. The 1,313 words not belonging to the stoplist were associated with each other based on the stem of each word, obtaining a final list of 607 stems (S1 Table).”

However, it is still unclear if it is a process they did by a Self-developed software. In this case, I suggest the authors indicate the software used and the source.

- **RP3**: Thank you for your comment. We extracted the stems manually. The main has been modified as follows:

**“**Then, to facilitate the analysis, the stemming procedure (manually conducted and not supported by software) was used to reduce inflected (or derived) words to their word stem (i.e., the main part of a word that stays the same when endings are added to it).”

10. **R3**: “Regarding the TF-IDF formula, the authors have specified which elements are used for the computation and clarified point by point the computational process. I believe that it is now more understandable, even for a non-expert reader, how the TF-IDF has been calculated, thus making the reproducibility of the results possible as well.”
    - **RP3**: We are happy to hear that the manuscript is now more understandable and the experiment reproducible thanks also to your comments.

References

Jones, M. C., Rosco, J. F., & Pewsey, A. (2011). Skewness-invariant measures of kurtosis. *The American Statistician*, *65*(2), 89–95. <https://doi.org/10.1198/tast.2011.10194>

Kenney, J. F., & Keeping, E. S. (1966). *Mathematics of statistics*. Van Nostrand Company.
